# The human ATAD5 has evolved unique structural elements to function exclusively as a PCNA unloader

**DOI:** 10.1038/s41594-024-01332-4

**Published:** 2024-06-13

**Authors:** Feng Wang, Qing He, Nina Y. Yao, Michael E. O’Donnell, Huilin Li

**Affiliations:** 1 Department of Structural Biology, Van Andel Institute, Grand Rapids, MI USA; 2 DNA Replication Laboratory and Howard Hughes Medical Institute, The Rockefeller University, New York, NY USA

**Keywords:** Cryoelectron microscopy, Enzyme mechanisms, Replisome, DNA replication

## Abstract

Humans have three different proliferating cell nuclear antigen (PCNA) clamp-loading complexes: RFC and CTF18-RFC load PCNA onto DNA, but ATAD5-RFC can only unload PCNA from DNA. The underlying structural basis of ATAD5-RFC unloading is unknown. We show here that ATAD5 has two unique locking loops that appear to tie the complex into a rigid structure, and together with a domain that plugs the DNA-binding chamber, prevent conformation changes required for DNA binding, likely explaining why ATAD5-RFC is exclusively a PCNA unloader. These features are conserved in the yeast PCNA unloader Elg1-RFC. We observe intermediates in which PCNA bound to ATAD5-RFC exists as a closed planar ring, a cracked spiral or a gapped spiral. Surprisingly, ATAD5-RFC can open a PCNA gap between PCNA protomers 2 and 3, different from the PCNA protomers 1 and 3 gap observed in all previously characterized clamp loaders.

## Abstract (web summary)

Cryo-EM structures of the human clamp unloader ATAD5-RFC bound to the sliding clamp PCNA reveal two unique locking loops and one chamber plug that prevent DNA from entering the ATAD5-RFC and explain why ATAD5-RFC is exclusively a PCNA unloader.

## Main

The eukaryotic DNA clamp is PCNA, a trimeric ring structure that encircles and slides along double-stranded DNA to promote processive DNA synthesis^1–4^. However, PCNA forms a central hub that regulates a wide range of cellular processes that extend well beyond DNA replication and include cell-cycle control, nucleotide excision repair, break-induced replication mismatch repair and chromatin assembly^5–8^. Owing to its closed topology, the PCNA ring needs to be opened and then ‘loaded’ onto the DNA by a dedicated ATPase machine. Upon completion of its function, the ring needs to be re-opened and ‘unloaded’ off the DNA by another molecular machine driven by ATP hydrolysis^9–11^. In replication, rapid recycling of PCNA from the replicated DNA to another single stranded–double stranded (ss/ds) DNA junction is essential to synthesize more than ten million Okazaki fragments, each of which is ~200 nucleotides (nt) long, in a human cell^11,12^.

**Fig. 1:**
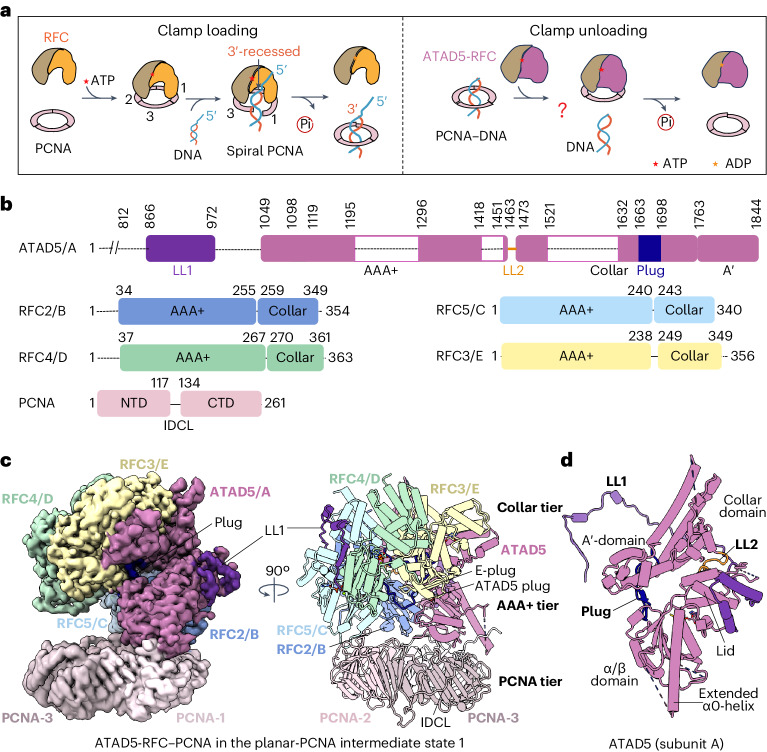
Cryo-EM structure of the human ATAD5-RFC–PCNA complex. **a**, The human PCNA clamp is loaded onto DNA by RFC (left) and unloaded from DNA by ATAD5-RFC (right). Although PCNA loading by RFC is well established, how ATAD5-RFC unloads PCNA from DNA and how ATAD5-RFC dissociates from unloaded PCNA have been unclear. **b**, Domain architectures of ATAD5, RFC2–RFC5 and PCNA. The five subunits in ATAD5-RFC are also labeled A to E. The two locking loops (LL1 in purple and LL2 in orange) and an insertion plug are highlighted. **c**, EM map (postprocessed with DeepEMhancer^44^) and atomic model of the ATAD5-RFC bound to a closed PCNA ring; individual subunits are colored. The ATAD5 LL1 and plug are highlighted in purple and blue, respectively. The three PCNA molecules are numbered based on the direction of their contact with subunits A to E in RFC and ATAD5-RFC. **d**, Structure of the largest subunit of ATAD5 (subunit A), with domains and key features labeled.

Human cells possess a canonical clamp loader—the pentameric replication factor C (RFC; RFC1–RFC5)—and three alternative clamp loaders, RAD17-RFC, CTF18-RFC and ATAD5-RFC^13–15^, which have different compositions; the largest subunit, RFC1, in RFC is replaced with RAD17, CTF18 or ATAD5, respectively. The clamp-loading process is mediated by three major clamp-loading complexes: RFC, CTF18-RFC and RAD17-RFC^15–17^. RFC is the most extensively studied^18–21^; binding of ATP to RFC leads to opening of the A-gate in RFC as well as binding and opening of the PCNA ring, which enables the 3′-ss/ds DNA junction to enter the RFC pentamer (that is, the ‘central chamber’) and the dsDNA region to enter the PCNA ring. Subsequent ATP hydrolysis by RFC leads to dissociation of RFC from PCNA, leaving PCNA to encircle the DNA^18^ (Fig. 1a). CTF18-RFC has an established role in sister chromatid cohesion^22^, and the complex has recently been proposed to load PCNA onto DNA specifically for the leading-strand replication by DNA polymerase epsilon^23,24^. Human RAD17-RFC and its yeast homolog, Rad24-RFC, load the heterotrimeric 9-1-1 ring onto a 5′-ss/ds DNA junction, a DNA clamp primarily involved in modulating kinase function in the DNA-damage cell-cycle checkpoint pathway^25–28^.

The PCNA-unloading process is not well understood. It has been reported that the PCNA loaders also unload PCNA in vitro, although unloading activity is much lower than loading activity^23,29^. Among the RFC-like complexes, only ATAD5-RFC and the yeast homolog Elg1-RFC have been found to exhibit potent PCNA-unloading activity^16^, but the underlying mechanism has been unclear. ATAD5 is a large protein with 1,844 amino acids (aa) that comprises two regions: a highly conserved amino-terminal region (1–600 aa) that binds the ubiquitin-specific-protease–USP1-associated factor 1 (USP–UAF1) complex to facilitate de-ubiquitination of ubiquitinated PCNA (Ub-PCNA)^30,31^, and a main body region (812–1844 aa) containing an AAA+ ATPase domain that is crucial for PCNA-unloading activity. Previous studies on the pentameric ATAD5-RFC complex revealed that it unloads PCNA much more efficiently than does the RFC complex^16,32,33^. Furthermore, knockdown of ATAD5 leads to prolonged PCNA retention on chromatin and an extended lifespan for DNA-replication factories^10,11,31,34–37^. Single-molecule experiments have revealed that ATAD5-RFC binds to a DNA-loaded PCNA to induce opening of the PCNA ring, leading to the release of ATAD5-RFC–PCNA from DNA^16^.

To understand why ATAD5-RFC acts only as a PCNA unloader, we purified N-terminal-truncated human ATAD5-RFC, which exhibits fully functional clamp unloading in vitro, and performed comprehensive cryo-electron microscopy (cryo-EM) analysis on the mixtures of ATAD5, PCNA and DNA in the presence of the weakly hydrolyzable ATP analog ATPγS. We obtained four cryo-EM structures of the ATAD5-RFC–PCNA complex (intermediates states 1–3 and 3′), in which PCNA is in different configurations that affect the ring opening and closing process during PCNA unloading or during dissociation of ATAD5-RFC from PCNA after unloading. We found that ATAD5 has evolved unique ‘locking loops’ on the outside and a ‘plug’ that fills the inner chamber; together, these seem to tie the ATAD5-RFC complex into a rigid structure that is compatible with PCNA binding and ring opening (for example, to exclude DNA from the opened ring), but is incompatible with loading PCNA onto DNA because the rigid complex and plug prevent DNA from entering the internal chamber. These structural features explain why ATAD5-RFC only unloads PCNA, distinguishing it from the other RFC-like complexes that primarily load PCNA.

## Results

### Structure of the human ATAD5-RFC bound to a closed-ring PCNA

**Extended Data Fig. 1:**
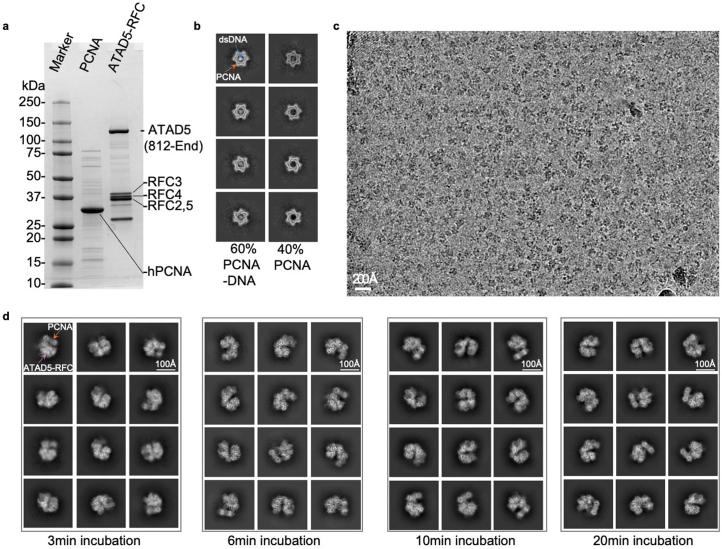
Cryo-EM analysis of purified human ATAD5-RFC bound to PCNA. **a**, SDS-PAGE gel of purified human PCNA (lane 2) and ATAD5-RFC (lane 3). The N-terminal 811 residues were truncated in the largest subunit ATAD5. This experiment was done once. **b**, Selected 2D class averages of the pre-assembled PCNA clamp and DNA complex by mixing the purified human PCNA and the DNA substrate. **c**, A typical raw micrograph of the in vitro assembled ATAD5-RFC–DNA–PCNA complex. A total of 18,828 such raw micrographs were recorded. **d**, Selected 2D class averages of four reaction mixtures incubated for either 3, 6, 10, or 20 min. The images demonstrate the interaction between PCNA and ATAD5-RFC, and despite the addition of DNA in the mixtures, no DNA was bound to these complexes. Source data

**Extended Data Fig. 2:**
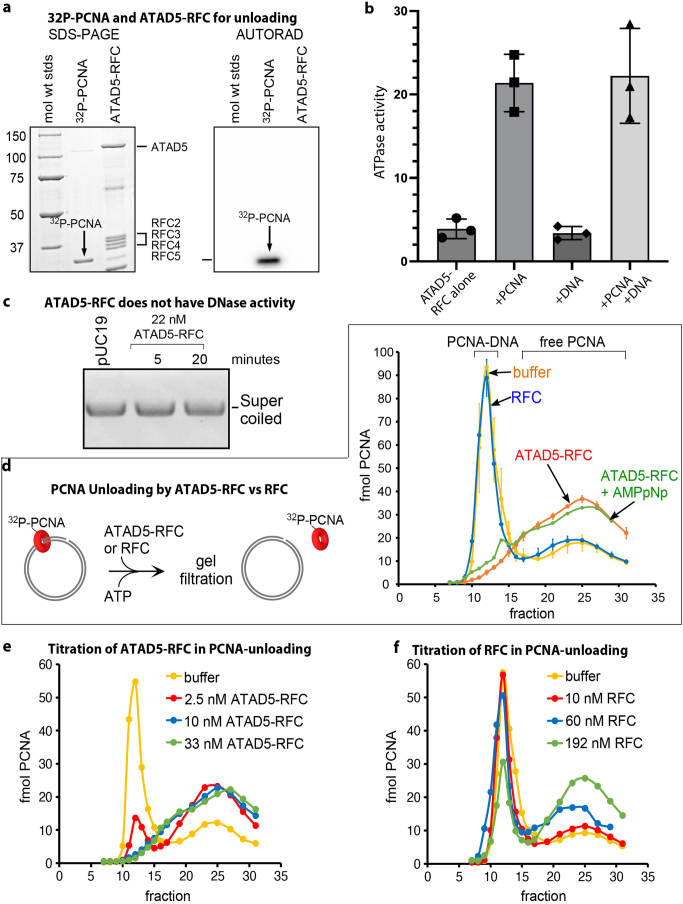
ATAD5-RFC ATPase and unloading activities. **a**, 8% SDS-PAGE of PCNA and ATAD5-RFC. Autoradiography of the PAGE on the right, showing 32P-labeling of PCNA. This experiment was done once. **b**, ATP hydrolyzed/min by ATAD5-RFC alone or with PCNA and/or DNA. Average activity ± s.e.m. is shown for n = 3 independent experiments. **c**, Native agarose gel of pUC19 stained with ethidium bromide, showing that ATAD5-RFC did not linearize or degrade the DNA. This experiment was done once. **d**, 10 nM ATAD5-RFC (red) or RFC (blue) was incubated with 32P-PCNA-DNA and 2 mM ATP for 5 min, followed by gel filtration to determine whether the 32P-PCNA is unloaded from DNA. 2 mM AMPPNP supported PCNA unloading by ATAD5-RFC (green). Experiments were performed in duplicate, and the data points are the average of two determinations performed on different days. The yellow line is a buffer control. See Methods for details. **e**, Titration of ATAD5-RFC in the unloading of 32P-PCNA from DNA. **f**, Titration of RFC in the unloading of 32P-PCNA from DNA. Source data

We purified the human ATAD5-RFC complexes by co-expressing in insect cells the N-terminal-truncated ATAD5 (aa 812–end) and the human RFC2–RFC5 subcomplex (Fig. 1b and Extended Data Fig. 1a). The N-terminal-truncated ATAD5 comprises a minimal core that is capable of unloading PCNA from DNA^31^. In the rest of the paper, we refer to the N-terminal-truncated ATAD5 as ATAD5. Purified ATAD5-RFC could unload PCNA and was approximately 100-fold more efficient than was canonical RFC in PCNA unloading (Extended Data Fig. 2a,c–f). Moreover, the non-hydrolyzable ATP analog AMPPNP supported the unloading activity of ATAD5-RFC (Extended Data Fig. 2d), indicating that ATP binding, but not ATP hydrolysis, is required for unloading activity, consistent with an earlier study using weakly hydrolyzable ATPγS^16^.

**Extended Data Fig. 3:**
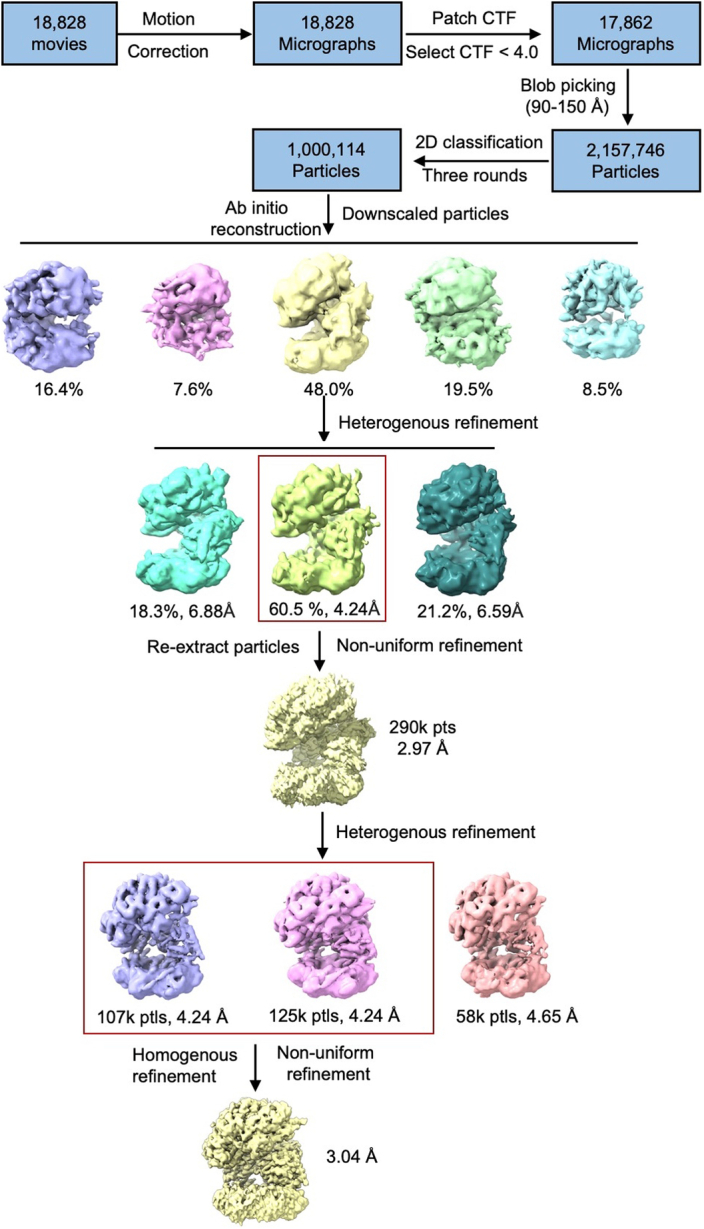
Workflow of cryo-EM data processing and 3D reconstruction of ATAD5-RFC bound to the closed-ring PCNA. CryoSPARC (version 3.2.0) was used for image processing and 3D reconstruction, yielding the final EM map at 3.04 Å average resolution.

**Extended Data Fig. 4:**
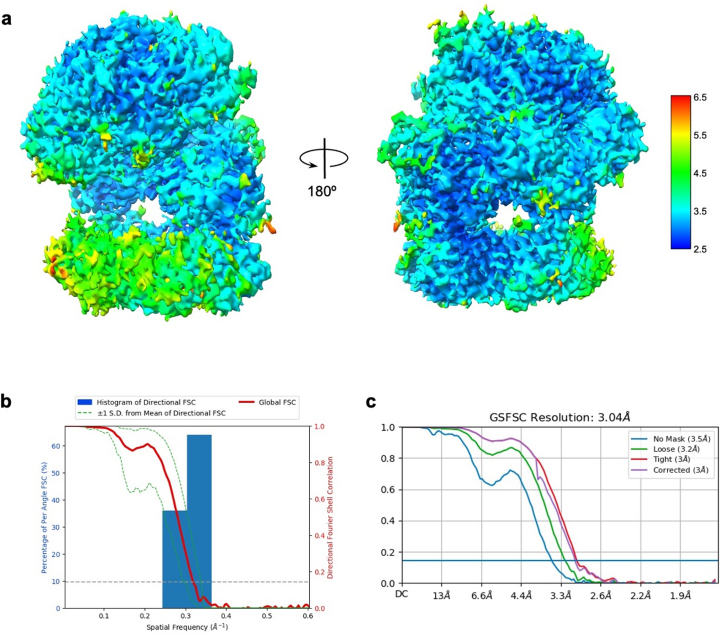
Resolution estimation of the 3D map of ATAD5-RFC-closed PCNA. **a**, Color-coded local resolution map of the ATAD5-RFC-closed PCNA EM map. **b**, The directional anisotropy of the ATAD5-RFC-closed PCNA 3D map as quantified by the 3D-FSC server (https://3dfsc.salk.edu/). The 3D map has a good anisotropic property with a sphericity of 0.891. **c**, The gold standard Fourier shell correlation curves of the final 3D map.

**Extended Data Fig. 5:**
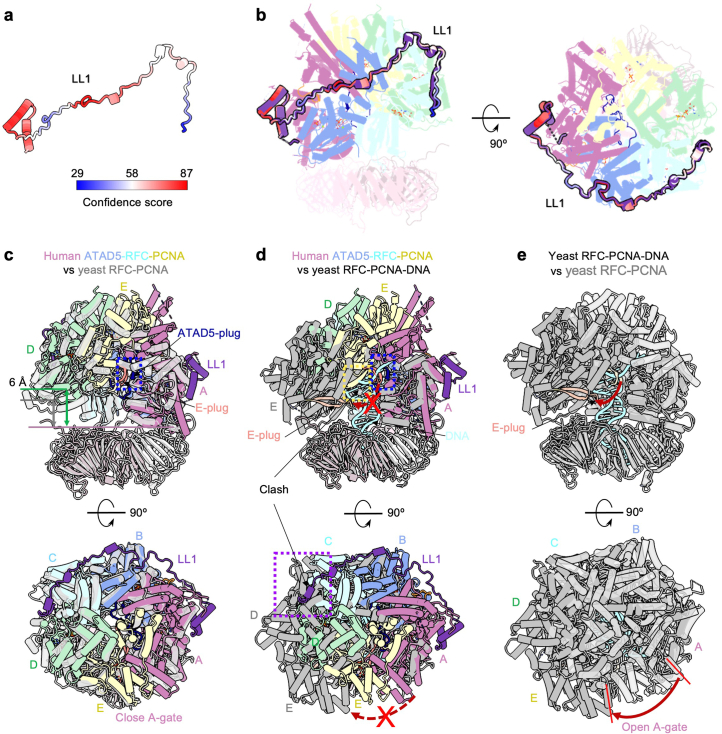
Comparison of ATAD5-RFC–PCNA with Alpha-fold2 predicted model regions and yeast RFC–PCNA–(DNA) structures. **a**, The Alpha-Fold2 predicted ATAD5 LL1 model colored by the prediction confidence score per residue. **b**, Superimposition of the predicted LL1 model with the cryo-EM structure, revealing their high similarity. **c**, Superimposition of human ATAD5-RFC-PCNA (color) with yeast RFC–PCNA (gray, PDB ID 7THI) in a front side (upper) and top view (lower). LL1 interacts with subunit D of ATAD5-RFC and moves up the subunit by 6 Å compared to the yeast RFC-PCNA. **d**, Superimposition of ATAD5-RFC–PCNA (color) with yeast RFC–PCNA–DNA (dark gray, dsDNA in cyan, PDB ID 7TID). The plug loop (blue) stabilizes the E-plug of ATAD5-RFC and sterically clashes with DNA in yeast RFC–PCNA–DNA. LL1 sterically clashes with subunit C of yeast RFC-PCNA-DNA. Unlike in yeast RFC–PCNA–DNA, the ATAD5-RFC E-plug cannot twist and the A-gate cannot open. **e**, Superimposition of yeast RFC–PCNA–DNA with yeast RFC–PCNA, showcasing the E-plug movement and the A-gate opening as indicated by red arrows.

To assemble in vitro a complex of ATAD5-RFC bound to PCNA encircling dsDNA, we mixed purified ATAD5-RFC complex (Fig. 1b and Extended Data Fig. 1a) with PCNA that was preassembled with a 38-base-pair (bp) dsDNA in the presence of 1 mM ATPγS, a slowly hydrolyzable ATP analog, and incubated the mixture for 20 min. The PCNA-encircled DNA complex was preassembled following an established procedure^38^, which yielded around 60% PCNA–DNA complex in the mixture (Extended Data Fig. 1b). We then prepared cryo-EM grids and recorded a cryo-EM dataset. Two-dimensional (2D) and three-dimensional (3D) classifications resulted in an EM map at an overall resolution of 3.0 Å (Fig. 1c, Extended Data Figs. 1c,d, 3 and 4 and Table 1). The high-quality EM map allowed us to build an atomic model for most regions of ATAD5-RFC–PCNA, except for several disordered loops (Fig. 1b,d). AlphaFold-Multimer was used to predict the long N-terminal loop in ATAD5. The predicted model had a high confidence level in most loop regions and was consistent with the final atomic model (Extended Data Fig. 5a,b).

**Table 1:** Cryo-EM data collection, refinement and validation statistics

	Intermediate state 1 of ATAD5-RFC–closed PCNA (EMD-42295), (PDB 8UII)	Intermediate state 2 of ATAD5-RFC–cracked spiral PCNA (EMD-42289), (PDB 8UI9)	Intermediate state 3 of ATAD5-RFC–gapped PCNA (EMD-42288), (PDB 8UI8)	Intermediate state 3′ of ATAD5-RFC–gapped PCNA (EMD-42287), (PDB 8UI7)
**Data collection and processing**				
Magnification	×105,000	×105,000	×105,000	
Voltage (kV)	300	300	300	
Electron exposure (e^–^/Å)	60	60	60	
Defocus range (μm)	1.2–1.6	1.2–1.6	1.2–1.6	
Pixel size (Å)	0.828	0.828	0.828	
Symmetry imposed	*C*_1_	*C*_1_	*C*_1_	
Initial particle images (no.)	1,000,114	1,027,202	1,027,202	
Final particle images (no.)	232,350	112,347	327,653	55,855
Map resolution (Å)	3.04	3.48	3.10	4.20
FSC threshold	0.143	0.143	0.143	0.143
Map resolution range (Å)	2.5–7.0	2.9–9.0	2.5–8.0	3.5–13.0
**Refinement**				
Initial model used	AF-Q96QE3-F1, PDB: 6VVO	Intermediate state 1	Intermediate state 1	Intermediate state 1
Model resolution (Å)	3.3	3.8	3.2	6.4
FSC threshold	0.5	0.5	0.5	0.5
Model resolution range (Å)	2.5–7.0	2.9–9.0	2.9–9.0	3.5–13.0
Map sharpening *B* factor (Å)	–108.1	–114.1	–133.3	–152.8
Model composition				
Non-hydrogen atoms	21,704	21,836	19,822	21,831
Protein / DNA residues	2,743 / 0	2,760 / 0	2,499 / 0	2,760 / 0
Ligands	9	9	8	8
*B* factors (Å^2^)				
Protein / DNA	73.36 / 0	111.32 / 0	80.75 / 0	253.54 / 0
Ligand	53.04	94.57	66.25	208.94
R.m.s. deviations				
Bond lengths (Å)	0.003	0.005	0.006	0.004
Bond angles (°)	0.758	0.883	1.142	0.969
Validation				
MolProbity score	1.51	1.64	1.52	1.76
Clashscore	7.23	8.21	7.70	10.63
Poor rotamers (%)	0.70	0.78	0.91	0.04
Ramachandran plot				
Favored (%)	97.46	96.78	97.54	96.60
Allowed (%)	2.54	3.22	2.46	3.37
Disallowed (%)	0	0	0	0.04

**Extended Data Fig. 6:**
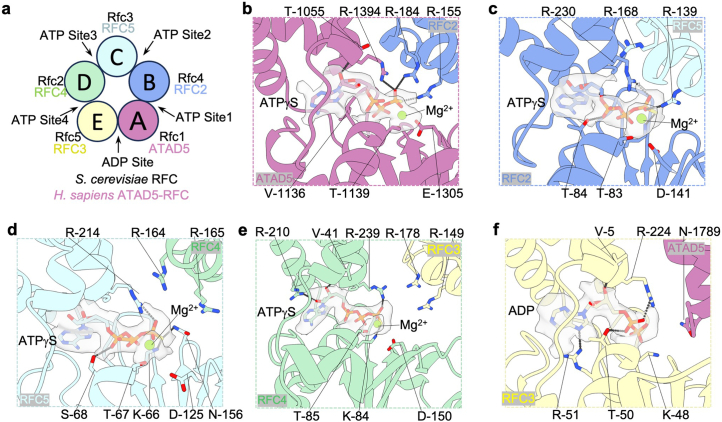
Subunit arrangement and the nucleotide binding in intermediate state 1 of the ATAD5-RFC–PCNA complex. **a**, The arrangement of human ATAD5-RFC subunits as compared to that of the yeast RFC, showing different numbering scheme between the yeast and human PCNA loaders. Nucleotide binding sites at subunit interfaces are indicated. **b-f**, Detailed view of the nucleotide-binding site in RFC subunit A/ATAD5 (b), B/RFC2 (c), C/RFC5 (d), and D/RFC4 (e) and E/RFC3 (f) of the ATAD5-RFC-closed PCNA complex. The bound ATPγS or ADP are shown in sticks with their respective EM density superimposed in semitransparent gray surface. The Mg^2+^ ion resolved in subunits A-D are in green spheres. The EM densities for the five bound nucleotides are surface rendered at a same threshold. Residues coordinating the nucleotides are in sticks and labeled.

In the ATAD5-RFC–PCNA complex, ATAD5-RFC sits above the PCNA ring, forming a three-tiered structure: ATAD5-RFC comprises the top collar tier and middle AAA+ tier, and the PCNA ring forms the bottom tier (Fig. 1d). The collar and AAA+ tiers are formed by the respective collar domains and AAA+ modules of ATAD5 and RFC2–RFC5 subunits arranged in a right-handed spiral. Because human RFC and yeast RFC were purified before structure determination and gene sequencing, the convention of numbering complex subunits by their respective size in a polyacrylamide gel resulted in the small RFC subunits having a different numbering scheme between yeast and human RFC. Thus, we also use the uniform convention of referring to clamp-loader subunits alphabetically, A, B, C, D and E, arranged counterclockwise when viewed from the carboxy-terminal collar domains. Thus, for human and yeast RFC, the subunits are A (hRFC1, yRfc1), B (hRFC2, yRfc4), C (hRFC5, yRfc3), D (hRFC4, yRfc2) and E (hRFC3, yRfc5) (Extended Data Fig. 6a). For ATAD5-RFC, the A subunit is ATAD5, which replaces RFC1. We found that four subunits (ATAD5, RFC2, RFC5 and RFC4, that is, subunits A–D) each bound an ATPγS, and RFC3 (subunit E) bound ADP (Extended Data Fig. 6b–f). The overall architecture and the nucleotide-binding pattern are shared with all reported clamp–clamp loader complex structures in the absence of DNA—from T4 phage to *Escherichia coli*, yeast and human^15,19–21,39,40^, and are similar to the 9-1-1 clamp loaders RAD17-RFC and Rad24-RFC^25–27^.

**Extended Data Fig. 7:**
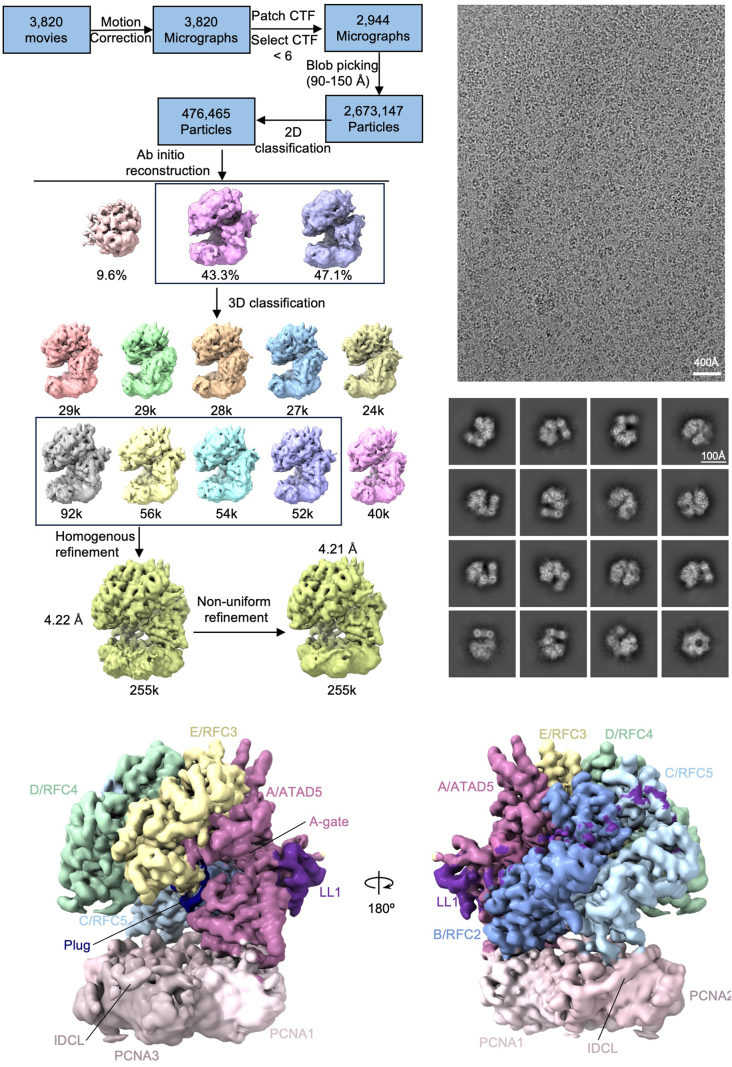
Cryo-EM of the ATAD5-RFC–PCNA complex obtained from mixture of ATAD5-RFC and PCNA in the absence of DNA. The EM map in the lower panels is indistinguishable from the EM map of the ATAD5-RFC–PCNA determined from the mixture in the presence of DNA as shown in Extended Data Fig. 3.

The middle AAA+ tier also contains the C-terminal A′ domain of ATAD5, which packs against the RFC3 AAA+ and forms the A-gate with the ATAD5 AAA+ module^18^ (Fig. 1d). The A-gate opens to admit the 3′-ss/ds DNA junction in a typical clamp loader, such as RFC^19–21^. We found that ATAD5 contains three unexpected features that are absent in any known clamp loader: an extended N-terminal loop, here called locking loop 1 (LL1), a second locking loop (LL2) and a plug (Fig. 1d); these elements seem to be crucial for ATAD5’s unloading function and will be discussed below. The structure is devoid of DNA, suggesting that, in our reaction, PCNA unloading by ATAD5-RFC was complete upon 20-min incubation, and PCNA returned to the closed planar ring in the complex (herein referred to as planar-PCNA intermediate state 1). However, although most PCNA rings (>60%) were bound to DNA before ATAD5-RFC was added to the mixture, it remains possible that ATAD5-RFC binds to PCNA after it slides off DNA. Indeed, we obtained a similar 3D structure to planar-PCNA intermediate state 1 by directly incubating ATAD5-RFC with free PCNA (Extended Data Fig. 7).

### Two unique ATAD5-RFC locking loops keep the A-gate shut

**Fig. 2:**
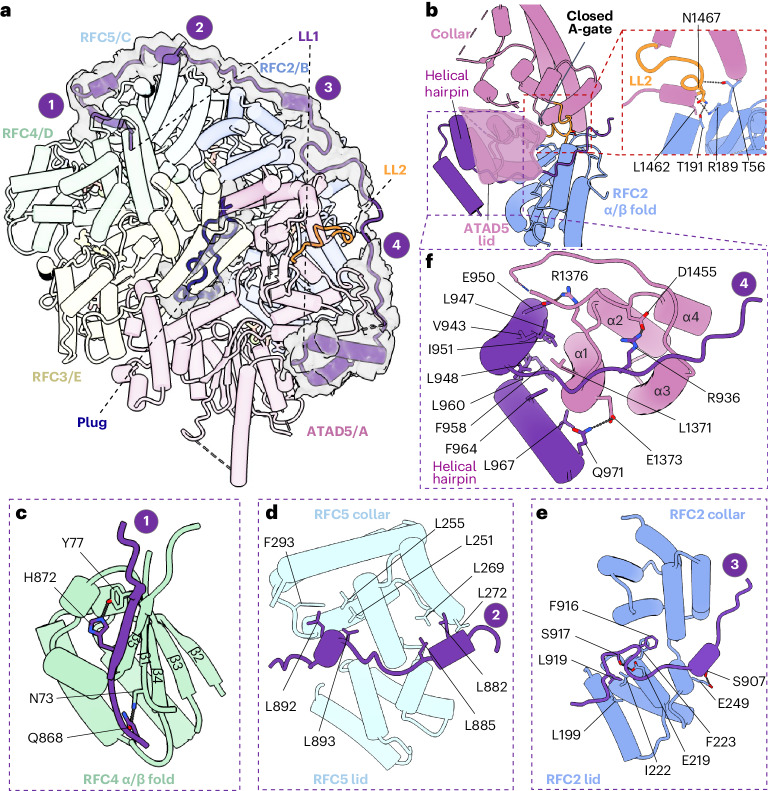
The ATAD5 LL1 locks the A-gate and prevents conformational changes in ATAD5-RFC. **a**, Top view of ATAD5-RFC. The bound nucleotide in each subunit is shown as sticks, and Mg^2+^ in spheres. The EM densities for LL1 and the plug are shown as transparent gray surfaces. LL1 is divided into numbered four regions, to better show detailed interactions in **c**-**e**. **b**, LL1 and LL2 lock the A-gate shut by stabilizing the ATAD5 lid and collar. The zoomed inset shows detailed interactions of LL2 with the RFC2 α/β fold. **c**-**e**, Close-up views of the first to third regions in LL1 interacting with RFC4 (**c**), RFC5 (**b**) and RFC2 (**e**). **f**, Close-up view of the fourth region of LL1 (the helix hairpin) interacting with the ATAD5 lid domain. Key interacting residues are shown as sticks and labeled. H-bonds are shown as dashed lines.

For a PCNA loader, A-gate opening is a crucial step that results in opening of the PCNA ring, allowing DNA access to both PCNA and the central chamber of the loader^19–21^. As mentioned above, ATAD5 contains three unique features that are not present in human RFC1 (Fig. 1d). The first unique feature is LL1, which is 105 residues long (Gln868 to Ala1072) and wraps around the outer surface of the unloader complex, meandering around ATAD5 (subunit A), RFC2 (subunit B) and RFC5 (subunit C). It then inserts into a cleft between RFC5 (subunit C) and RFC4 (subunit D) (Fig. 2a). LL1 was well resolved in the EM map, and could be divided into four regions (Fig. 2a). Local interactions convert several LL1 regions into α-helices and β-strands. Indeed, LL1 adopts essentially the same conformation as the other conformers (which are discussed later). An earlier study showed that substitution of four residues in LL1 (872–875 aa) with four alanines severely decreased ATAD5-RFC unloading action in cells, consistent with the importance of LL1 for ATAD5-RFC function^31^. The second unique feature is that the alternative linker (AL) loop between the collar and the AAA+ module of ATAD5, which is conserved among the RFC loaders and unloaders, is positioned right above the A-gate and near LL1, suggesting that the AL has a role in locking the A-gate shut. We therefore renamed the AL loop in ATAD5 to locking loop 2 (LL2). LL2 forms three H-bonds with the RFC2 α/β subdomain: Asn1467 with RFC2 Thr191, the main chain oxygen of Asn1467 with RFC2 Thr56, and the main chain oxygen of Leu1462 with RFC2 Arg189. These H-bonds stabilize LL2 firming its apparent action as a lock that blocks the ATAD5 A-gate opening (Fig. 2b). In comparison, the RFC1 AL is flexible and enables A-gate opening^18,21^. The third unique feature, to be discussed in detail below, is the ATAD5 plug, comprising 36 residues (Leu1663–Asp1698), that occupies the central chamber (Fig. 2a).

The LL1 region 1 folds into a β-strand to complement the five-stranded β-sheet core (β-1–β-5) of the RFC4 α/β subdomain (Fig. 2c). Thus, this LL1 region is tightly coupled to the RFC4 α/β fold, and they can move together as a rigid body. The LL1 β-strand is parallel to the adjacent β5 and forms two additional H-bonds with the β1-strand: LL1 Gln868 with β1 Gln73, and LL1 His872 with β1 Tyr77. The LL1 region 2 is hydrophobic; it contains two short α-helices and wraps around the side of the RFC5 collar domain, in which LL1 residues Leu882, Leu885, Leu892 and Leu893 form hydrophobic interactions with the RFC5 collar residues Leu269, Leu272, Leu251, Leu255 and Phe293 (Fig. 2d). The LL1 region 3 is inserted into a cleft between the lid and collar domains of RFC2 and interacts primarily with the RFC2 lid (Fig. 2e): LL1 Leu919 forms a hydrophobic interaction with the RFC2 lid residues Leu199 and Ile222, LL1 Phe916 forms a π–π interaction with the RFC2 lid residues Phe223 and Ser907 and LL1 Ser917 forms an H-bond with the RFC2 lid residues Glu249 and Glu219. The LL1 region 4 contains a helix hairpin that, together with LL2, sandwiches the ATAD5 helical lid domain and holds the lid close to the RFC2 α/β subdomain, thereby preventing the A-gate from opening (Fig. 2b). Specifically, the helix hairpin of LL1 region 4 packs tightly against the ATAD5 lid domain, involving eight hydrophobic residues (Val943, Leu947, Leu948, Ile951, Phe958, Leu960, Phe964 and Leu967) (Fig. 2f). The lid α1-helix wedges into the hydrophobic cleft of the helix hairpin and contributes Leu1371 to the interaction. The LL1 region 4 also forms three H-bonds with the lid: Glu950 with lid Arg1376, Gln971 with lid Glu1373 and Arg936 with lid Asp1455. In summary, the extensive interactions stabilize the LL1, and in turn, the LL1 seems to tie the ATAD5-RFC into a stable structure that is resistant to the conformational changes that are required for binding of the same substrate (for example, ssDNA, dsDNA, gap, nick) as the clamp loaders in the central cavity^18–21^.

### The ATAD5 plug prevents DNA from entering the ATAD5-RFC

**Fig. 3:**
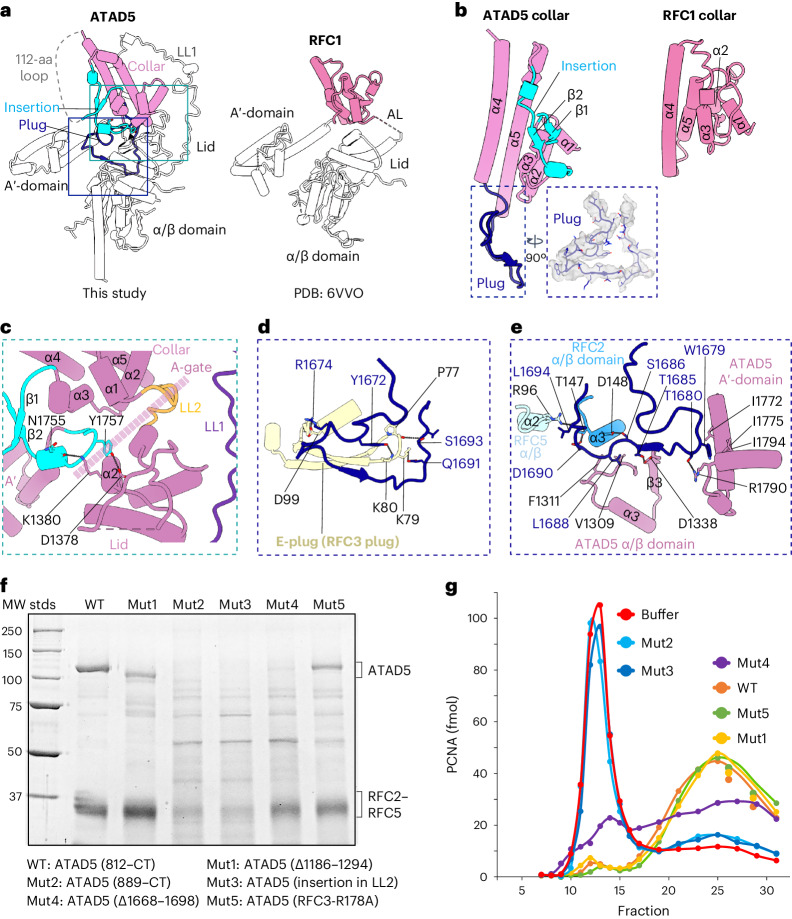
The unique ATAD5 plug occupies the central chamber of the unloader complex. **a**, Side-by-side comparison of human ATAD5 and RFC1 (PDB 6VVO). The collar domain is colored, and all other regions are shown in light gray. The plug and associated insertion regions in the ATAD5 collar domain are colored blue and cyan, respectively. The disordered 112-aa connecting loop is shown by a dashed cyan line. **b**, Comparison of the collar domains of ATAD5 (left) and RFC1 (right). The conserved α-helices in both collar domains are labeled. Bottom: the ATAD5 plug is shown as sticks and is superimposed with the EM density in semitransparent gray surface view. **c**, Close-up of the cyan box in **a**, showing the ATAD5-plug-associated insertion region near the closed A-gate. The insertion region interacts with the ATAD5 lid domain to help LL1 and LL2 lock the A-gate. Key residues in the interaction are shown as sticks and labeled. **d**,**e**, Two close-up views of the blue box in **a**, in the context of the ATAD5-RFC pentamer. The ATAD5 plug forms a short antiparallel β-sheet with the conserved E-plug (**d**) and is stabilized by the AAA+ domains of RFC2, RFC3 and RFC5 (**d**,**e**). The ATAD5 plug also bridges the α/β fold and the A′ domain to rigidify ATAD5 (**e**). H-bonds are shown as dashed lines, and key interacting residues are shown in sticks and labeled. **f**, SDS–PAGE (8%) of 4 μg each of WT ATAD5-RFC and the five ATAD5-RFC mutants (Mut1–Mut5). Mut1–Mut4 contain either deletions or insertions in the truncated ATAD5 (Δ1–812). Mut5 has an amino acid replacement in the Arg finger residue of RFC3. The gel analysis was performed once. The label ‘MW stds’ refers to molecular weight standards. CT, C terminus. **g**, Comparison of unloading activity of WT ATAD5-RFC with ATAD5-RFC mutants; 6.5 nM ATAD5-RFC (either WT or mutants) was incubated with ^32^P-labeled PCNA–DNA and 2 mM ATP for 5 min at 37 °C, followed by gel filtration to determine whether the ^32^P-labeled PCNA is unloaded from DNA. The *x* axis label ‘Fraction’ refers to individual gel filtration fractions collected for activity assay. This experiment was performed once. Source data

The remaining key feature of ATAD5-RFC is the ATAD5 plug inside the central chamber (Figs. 1d and 2a). The RFC1 collar domain contains five α-helices (α1–α5) and connects to the A′-domain through a short loop (Fig. 3a,b). The ATAD5 collar domain also contains five α-helices, but α2 and α5 are much longer than the corresponding helices in human RFC1 (Fig. 3b). The ATAD5 plug is formed through extension of the linker loop between the α4 and α5 helices of the ATAD5 collar domain, as compared with the human RFC1 collar domain (Fig. 3a,b). The ATAD5 collar contains two additional insertions: a 110-residue insertion between α3 and α4 containing a short β-stand (β1) and disordered remaining region, and another insertion between α5 and A′-domain containing another short β-stand (β2) (Fig. 3a,b). The short β-stands of the two insertions form a parallel two-stranded β-sheet. The two inserted regions interact with the α2 helix of the lid: Tyr1757 and the main chain oxygen of Asn1755 form H-bonds with the lid residues Asp1378 and Lys1380, respectively (Fig. 3c). These insertions glue the lid and A′-domain together, and function together with LL1 and LL2 to block the A-gate (Fig. 3c).

The ATAD5 plug contains a short β-strand that complements the two-strand E-plug (RFC3-Plug) (Fig. 3d). The E-plug is a conserved feature among all clamp loaders and unloaders: its two positively charged residues insert into the DNA main groove in all known clamp-loader structures^15^. However, in the ATAD5-RFC unloader, ATAD5 plug residues Gln1691 and Tyr1672 form H-bonds with E-plug residues Lys79 and Lys80, respectively, thereby neutralizing the E-plug’s ability to interact with DNA. The ATAD5 plug residues Arg1694 and Ser1693 form two additional H-bonds with Asp99 and the main chain oxygen of Pro77 in the E-plug, respectively (Fig. 3d).

The ATAD5 plug interacts with all ATAD5-RFC subunits, except for RFC4 (Figs. 1d and 3d,e). The ATAD5 plug residues Ser1686 and Asp1690 and the main chain oxygen of Leu1694 form H-bonds with Asp148 and Thr147 in RFC2 and Arg96 in RFC5, respectively (Fig. 3e). The ATAD5 plug also bridges the ATAD5 α/β domain and the A′-domains to rigidify the entire ATAD5 molecule. Specifically, Leu1688 in the ATAD5 plug interacts hydrophobically with Val1309 and Phe1311 in the α/β domain; Thr1685 forms an H-bond with Asp1338 in the α/β domain; Trp1679 inserts into a hydrophobic pocket in the A′-domain lined by Ile1772, Ile1775 and Ile1794; and Thr1680 forms an H-bond with the A′ domain Arg1790 (Fig. 3e). Therefore, the ATAD5 plug resides inside the central chamber and forms a number of interactions that rigidify the clamp-loader complex and fills its central chamber to the exclusion of DNA.

To functionally validate the unique structural features in ATAD5, we generated five ATAD5-RFC mutants (Mut1–Mut5) and performed the PCNA-unloading assay. In Mut1 (Δ1186–1294), the long flexible loop region of ATAD5 and part of the interface with PCNA was deleted; in Mut2 (Δ812–889), the LL1 was deleted; in Mut3, four residues (GGGS) were inserted in LL2; in Mut4 (Δ1668–1698), the A plug was deleted; and Mut5 had a p.R178A substitution in the RFC3 arginine finger region that inactivated the RFC4 ATPase site. Mut1 and Mut5 formed a stable ATAD5-RFC complex, and their PCNA-unloading activity was indistinguishable from that of the wild-type (WT) protein (Fig. 3f,g). However, Mut2, Mut3 and Mut4 were either not expressed well or were not stably incorporated into the RFC complex, indicating that LL1, LL2 and the plug have important roles in ATAD5-RFC structural integrity. Mut4 exhibited reduced assembly of the full complex than did the WT protein, with a corresponding reduction in unloading activity (Fig. 3f,g). This could simply reflect the lower concentration of full complex, considering that a reduction of WT ATAD5-RFC concentration from 10 nM to 2.5 nM led to a similar decrease in unloading efficiency (Extended Data Fig. 2e). Therefore, we cannot rule out the possibility that Mut4 is as active in unloading as the WT protein. In conclusion, owing to a loss of structural integrity of Mut2, Mut3 and Mut4, it remains unclear whether LL1, LL2 and the plug have a direct role in PCNA unloading.

### ATAD5 has a larger interface with PCNA than RFC1

Three subunits of ATAD5-RFC interact with two of the three PCNA subunits: ATAD5 (subunit A) and RFC2 (subunit B) contact PCNA-1, and RFC5 (subunit C) contacts PCNA-2 (Fig. 1c). Notably, no subunit contacts PCNA-3. The PCNA-binding interface of ATAD5 (subunit A) is larger than those of RFC2 and RFC5 (subunits B and C) (Fig. 1c). These interaction patterns resemble those in several recently determined RFC–PCNA structures in an autoinhibited state that occurs before RFC opens the PCNA ring for loading onto DNA^15,19,20^. However, ATAD5-RFC is a clamp-unloading complex and needs to first interact with the DNA-bound PCNA. Therefore, we propose that the closed planar-PCNA state represents a late intermediate stage in the unloading process in which ATAD5-RFC has unloaded the PCNA from DNA, and the PCNA ring has closed and is ready to dissociate from ATAD5-RFC, as explained later.

**Fig. 4:**
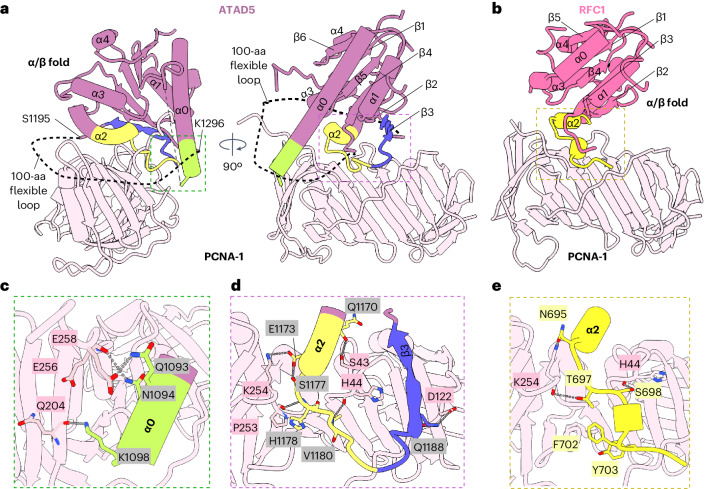
The AAA+ module of ATAD5 contains a PCNA-binding interface larger than that of RFC1. **a**, The interface between ATAD5 and PCNA-1. The ATAD5 non-canonical PIP box is colored yellow, and the two additional PCNA-binding regions are colored mint green and blue. The secondary structures in the α/β fold are labeled. **b**, The interface between human RFC1 and PCNA-1 (PDB: 6VVO). RFC1 binds PCNA-1 by only the conserved PIP box (yellow). **c**, Close-up view of the interactions between the ATAD5 α0 and PCNA-1 (dashed green square in **a**). **d**, Close-up view of interactions of the ATAD5 PIP box and the extended β3-strand with PCNA-1 (dashed magenta square in **a**). **e**, Close-up view of the interactions of the conserved RFC1 PIP box with PCNA-1 (dashed yellow square in **b**). Residues involved in the interactions are shown in sticks and labeled in **c**-**e**.

A unique feature in ATAD5 is the much-extended α0-helix in the AAA+ domain, compared with that of human RFC1 (PDB: 6VVO)^15^ (Fig. 4a,b). The ATAD5 α0-helix is long enough to reach PCNA and interact with the PCNA-1 C terminus through three H-bonds between Gln1093 in α0 and PCNA Glu258, Gln1094 in α0 and the main chain oxygen of Glu256 in PCNA, and Lys1098 in α0 and the main chain oxygen of Gln204 in PCNA-1 (Fig. 4c). Furthermore, the α2 element of ATAD5 contains a non-consensus PCNA-interacting peptide (PIP) motif (1173-EATQSHQV-1180). The PIP motif inserts into the PIP pocket and forms four H-bonds with PCNA-1: the main chain oxygen of Gln1170 in the PIP motif with Ser43 in PCNA-1; Gln-1173 in the PIP motif with Lys254 in PCNA-1; the main chain nitrogen of His1178 in the PIP motif with the main chain oxygen of Pro253 in PCNA-1; and the main chain nitrogen of Val1180 in the PIP motif with the main chain oxygen of His44 in PCNA-1. Another unique feature in ATAD5 is an extra β-strand (β3) in the AAA+ domain following the PIP motif (Fig. 4d). ATAD5 β3 interacts with PCNA-1 through an additional H-bond between Gln1188 in the β3-strand and Asp122 in PCNA-1. These two unique features resulted in a large interface between ATAD5 and PCNA-1, spanning 1,295 Å^2^. In comparison, human RFC1 contains the classic PIP motif that inserts into the PIP pocket of PCNA-1, and forms hydrophobic interactions with the Phe702 and Tyr703 residues of PCNA-1 (ref. ^15^). Human RFC1 also forms three H-bonds with PCNA: Asn695 in RFC1 with Lys254 in PCNA-1; Thr697 in RFC1 with the main chain oxygen of Lys254 in PCNA-1; and Ser698 with the main chain oxygen of His44 in PCNA-1 (Fig. 4e). The interface between human RFC1 and PCNA-1 is 1,009 Å^2^. The significantly larger PCNA-binding interface between ATAD5 and PCNA (1,295 Å^2^) could contribute to ATAD5’s unloading activity. Finally, the ATAD5 AAA+ domain contains an unusually long 100-residue loop connecting the β3- and β4-strands. This loop is disordered in our structure, but it can’t thread through the small tunnel between the ATAD5 AAA+ domain and RFC2 (subunit B) and must wrap around the ATAD5 α/β subdomain, thereby passing through the central chamber of the ATAD5-RFC complex (Fig. 4a). This long loop could contact the inner face of the PCNA ring. However, deleting the flexible loop in the ATAD5 AAA+ domain (Mut1) did not markedly affect the PCNA-unloading activity by the unloader (Fig. 3f,g). Therefore, the function of the flexible loop is unclear.

### ATP hydrolysis by RFC4 might open PCNA

**Fig. 5:**
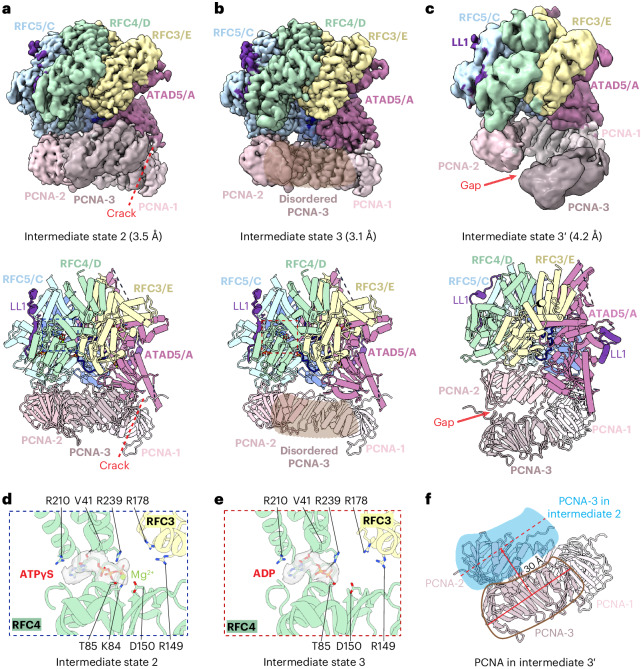
ATP hydrolysis by RFC4 underlies the transition of the PCNA crack from the right to the left of PCNA-3. **a**-**c**, EM maps (top; postprocessed with DeepEMhancer^44^) and atomic models (bottom) of ATAD5-RFC bound to PCNA in intermediate states 2 (**a**), 3 (**b**) and 3′ (**c**). Maps and structures are colored by individual subunits, as in Fig. 1b. **d**,**e**, Close-up views of the nucleotide-binding regions in RFC4 (subunit D) in intermediates 2 (**d**) and 3 (**e**), corresponding to areas marked by the blue and red squares at the bottom of **a** and **b**. The nucleotides and interacting residues are shown as sticks and labeled. **f**, The open (gapped) PCNA structure in the intermediate state 3′. The PCNA structure of intermediate state 2 is superimposed, but only the position of its PCNA-3 is shown (in cyan), to reveal a 30-Å movement of PCNA-3 between intermediate state 2 and intermediate state 3′.

**Extended Data Fig. 8:**
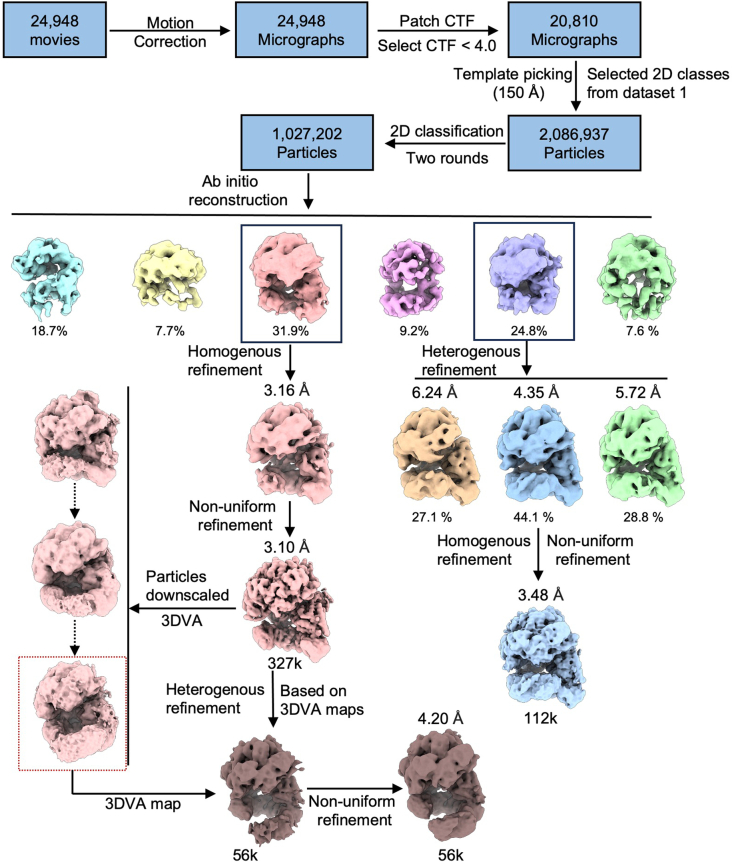
Workflow of cryo-EM data processing and 3D reconstruction of ATAD5-RFC–cracked PCNA. CryoSPARC (version 3.2.0) was used for processing this dataset, leading to an EM map for the ATAD5-RFC bound to a gapped PCNA ring at an average resolution of 3.10 Å and another EM map of ATAD5-RFC bound to a cracked PCNA ring at an overall resolution of 3.48 Å. 3DVA analysis of the particles belong to the gapped 3.10 Å map led to the capture of ATAD5-RFC bound to a cracked ring with a tilted PCNA-3 at an overall resolution of 4.20 Å.

**Extended Data Fig. 9:**
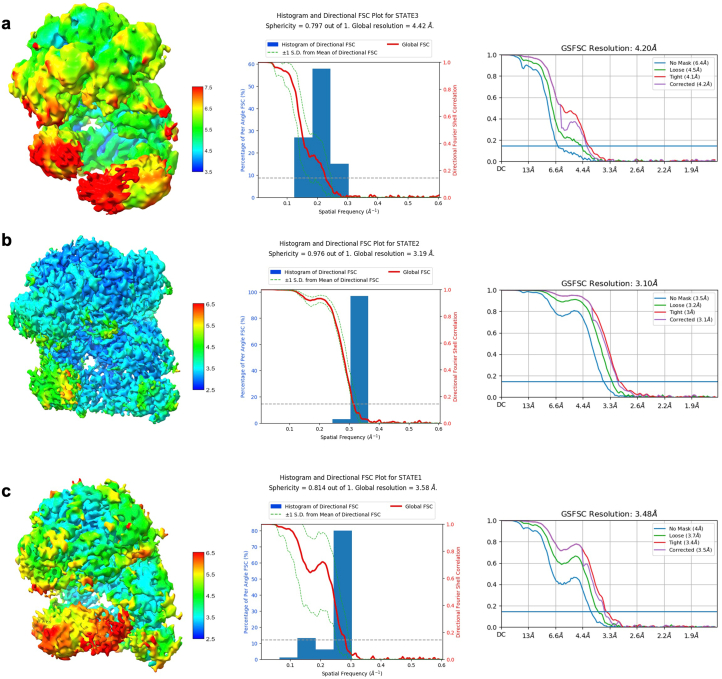
Local resolution estimation of the three EM maps of the ATAD5-RFC–PCNA. **a**, Resolution estimation of the ATAD5-RFC-cracked PCNA in intermediate state 3’. **b**, Resolution estimation of the ATAD5-RFC-cracked PCNA in intermediate state 3. **c**, Resolution estimation of the ATAD5-RFC-cracked PCNA in intermediate state 2. Left panels are color-coded local resolution map of the ATAD5-RFC-cracked PCNA EM map. Middle panels are directional anisotropy of the 3D map as measured by the 3D-FSC server (https://3dfsc.salk.edu/). Right panels are gold standard Fourier shell correlation curves of the three EM maps.

The structure in Fig. 1 (planar-PCNA intermediate state 1) was obtained from a reaction that lasted for 20 min. To look for earlier intermediates in the reaction, we next used cryo-EM to examine three assembly mixtures incubated for decreasing amounts of time, ranging from 10 to 6 to 3 min, to visualize the intermediates during ATAD5-RFC unloading of PCNA from DNA. We did not observe any complex bound to DNA in these samples (Extended Data Fig. 1d). However, we obtained from the 3-min incubation mixture two other intermediate states (cracked-PCNA intermediate 2 and disordered-PCNA intermediate 3) of the ATAD5-RFC–PCNA binary complex, at average resolutions of 3.5 Å and 3.1 Å, respectively (Fig. 5a,b, Table 1 and Extended Data Figs. 8 and 9). ATAD5-RFC does not undergo large conformational changes in these intermediates; in particular, the conformations of the unique features (such as LL1, LL2 and the plug) of ATAD5 in these states are the same as in the planar-PCNA intermediate state 1 that was obtained from the 20-min incubation sample. But the PCNA rings in these intermediate states are different from the planar-PCNA intermediate state 1 (Figs. 1c and 5a,b). In the cracked-PCNA intermediate state 2, all three PCNA molecules are ordered and form a right-handed spiral with a crack between PCNA subunits 1 and 3—but not a gap (Fig. 5a). In the disordered-PCNA intermediate state 3, PCNA-1 and PCNA-2 are well resolved, but PCNA-3 is disordered and invisible (Fig. 5b). We performed 3D variability analysis (3DVA) and found that the PCNA-3 undergoes a large-scale movement (Supplementary Video 1): PCNA-3 tilts down to form a cracked interface with PCNA-2 on the left, then slides upward to form a new cracked interface with PCNA-1 on the right. From the disordered-PCNA intermediate state 3 dataset, we derived another intermediate structure (open-PCNA intermediate state 3′) at an average resolution of 4.2 Å (Extended Data Fig. 8) that contains a 5-Å gap between PCNA-2 and PCNA-3 (Fig. 5c). This gap is unique because, to date, all structures of clamp loaders with open PCNA have a gap between PCNA-1 and PCNA-3.

**Extended Data Fig. 10:**
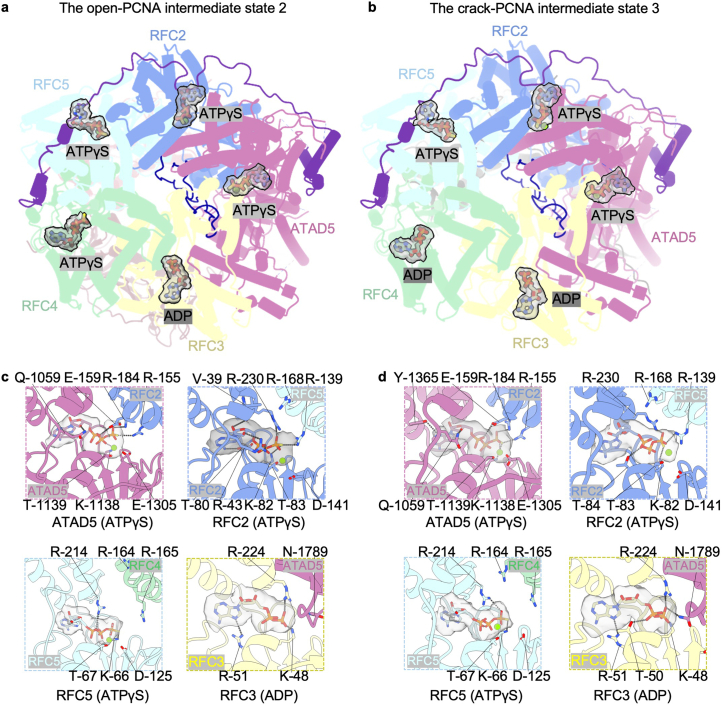
Nucleotide binding in ATAD5-RFC–PCNA in the cracked-PCNA intermediate state 2 and the open-PCNA intermediate state 3. **a-b**, Top views of the ATAD5-RFC in cracked-PCNA intermediate state 2 (**a**) and the open-PCNA intermediate state 3 (**b**). **c**-**d**, Detailed view of the nucleotide-binding site in ATAD5 and RFC2, 5, 3 in intermediate states 2 (**c**) and 3 (**d**). In all panels, the nucleotides are in sticks superimposed with the EM densities rendered at the same threshold in semi-transparent surfaces. And resolved Mg2+ ion in ATAD5 and RFC2, 5 are in green spheres. Residues interacting with the nucleotides are in sticks and labeled.

Unexpectedly, the RFC4 (subunit D) in the disordered-PCNA and open-PCNA intermediate states 3 and 3′ is occupied by ADP. The ATPγS in RFC4 of cracked-PCNA intermediate 2 is stabilized by Val41, Lys84 and an Mg^2+^ coordinated by Thr85 and Asp150. By contrast, the ADP in RFC4 of states 3 and 3′ is stabilized by Val41, Thr85 and Arg239 (Fig. 5d,e). Therefore, the ATPγS in RFC4 seems to have been either hydrolyzed to ADP or exchanged for ADP in the transition between the cracked-PCNA and disordered-PCNA intermediate states 2 and 3 (Fig. 5c,d and Extended Data Fig. 10). Superimposition of PCNA-1 in the cracked-PCNA and open-PCNA states shows that PCNA-3 moves by 30 Å (Fig. 5f). We suggest that the cracked versus open PCNA state is due to different nucleotide occupancies in these ATAD5-RFC intermediates.

What might these intermediate states suggest about the role of ATP hydrolysis? Although this report does not claim to describe the unloading mechanism of ATAD5-RFC, it sheds light on a possible function of hydrolysis. Consider the canonical RFC. RFC needs only to bind ATP to open or close PCNA around DNA; ATP hydrolysis is needed for RFC to eject from PCNA encircling DNA^18^. This is similar to ATAD5-RFC, which needs only to bind ATP to open PCNA for unloading (Extended Data Fig. 2d). Consistently, inactivation of RFC4 ATPase (Mut5, Arg finger mutant RFC3-R178A) did not affect PCNA-unloading activity (Fig. 3g). We therefore presume that, similar to RFC, ATAD5-RFC uses ATP hydrolysis to dissociate from PCNA, allowing the complex to be reused in the unloading of other PCNA clamps on DNA. This proposed action predicts that PCNA may alter the ATPase activity of ATAD5-RFC. Indeed, we found that PCNA stimulated the ATPase activity of ATAD5-RFC by fivefold (Extended Data Fig. 2b). However, DNA had no effect on the ATPase activity of ATAD5-RFC (Extended Data Fig. 2b), unlike RFC which is stimulated by DNA. These ATPase properties are consistent with those of yeast Elg1-RFC^23^.

### The RFC5 AAA+ module may control PCNA ring closure

**Fig. 6:**
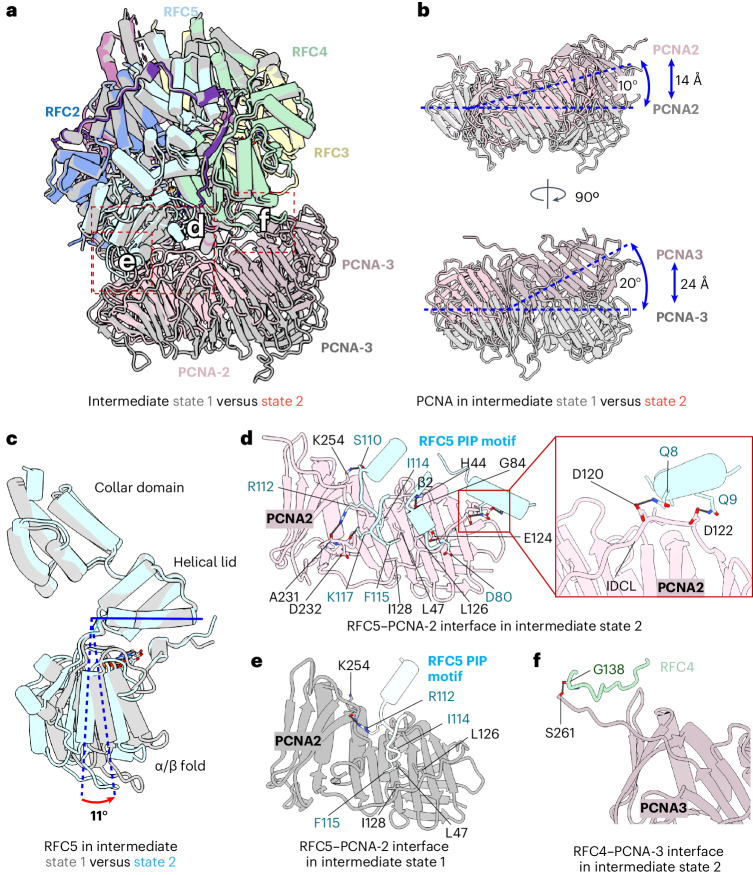
Conformational change in RFC5 underlies a lock-washer to planar-ring transition of PCNA. **a**, Superimposition of ATAD5-RFC–PCNA in intermediate states 1 and 2. Intermediate state 2 is shown in color, and state 1 in gray. **b**, Two side views of the superimposed PCNA structures showing that PCNA-2 and PCNA-3 undergo large rigid-body movements from a right-handed spiral to a planar ring. **c**, Overlay of RFC5 (subunit C) in intermediate state 2 (cyan) and in state 1 (gray). The α/β domain rotates 11° with respect to the helical lid. **d**–**f**, Close-up views of the three boxed regions in **a**, showing detailed interactions between the RFC5 PIP motif and PCNA-2 in intermediate state 2 (**d**), between the RFC5 PIP motif and PCNA-2 in intermediate state 1 (**e**) and between RFC4 and PCNA-3 in intermediate state 2 (**f**). The red box in **d** is enlarged on the right to show the RFC5 N terminus interacting with PCNA-2. Key residues are shown as sticks and labeled.

Comparing the closed-PCNA and cracked-PCNA intermediate states 1 and 2 reveals that both PCNA-2 and PCNA-3 undergo a large conformational change, in which PCNA-2 and PCNA-3 tilt by 10° and 20°, and their N-terminal halves move by 14 Å and 24 Å, respectively, with PCNA-1 remaining stationary (Fig. 6a,b). The planar-PCNA and cracked-PCNA intermediate states 1 and 2 were captured by incubating the reaction mixtures of ATAD5-RFC and PCNA–DNA for 20 min or 3 min, respectively. Their corresponding subunits are similar, with a small r.m.s. deviation (r.m.s.d.) (~0.6 Å) for ATAD5, RFC2, RFC4 and RFC3, and a much larger r.m.s.d. (1.2 Å) for RFC5. Superimposition of the two RFC5 structures shows that the α/β fold domain and the helical lid domain are orthogonal (90°) in the cracked-PCNA intermediate state 2 but are 79° apart in the planar-PCNA intermediate 1 (Fig. 6c). In the cracked-PCNA intermediate state 2, RFC5 interacts with PCNA-2 through three elements, leading to an extensive interface of 3,044 Å^2^ (Fig. 6d): the non-canonical PIP motif (Ser110 to Lys117), the α1-extension-helix and the N-terminal peptide (Met1 to Ala13). The RFC5 PIP residues Phe115 and Ile114 are inserted into the PCNA-2 PIP-binding cleft, lined by Leu47, Leu126 and Ile128. The RFC5 PIP motif also forms several H-bonds with PCNA-2: Ser110, Arg112 and Lys117 in the PIP motif form H-bonds with Lys254 and the main chain oxygens of Ala231 and Asp232 in PCNA-2. The α1-extension-helix forms two H-bonds with PCNA-2, between the main chain oxygen of Gly84 and Asp80 in RFC5 and His44 and Glu124 in PCNA-2, respectively. The RFC5 N-terminal α-helix forms two H-bonds with the PCNA-2 interdomain connector loop, between Gln8 and Gln9 in RFC5 and Asp122 and Asp120 in PCNA-2, respectively. In the planar-PCNA intermediate state 1, the interface is smaller (2,059 Å^2^; Fig. 6e): the aromatic residue Phe115 of the PIP box also inserts into the PIP-box binding cleft of PCNA-2, and Arg112 in the PIP motif forms a single H-bond with the main chain oxygen of Lys254 in PCNA-2. The RFC5 N-terminal α-helix and the α1-extension-helix move up and lose contact with PCNA-2. Additionally, RFC4 interacts with the PCNA-3 C-terminal loop through an H-bond between Gly138 in RFC5 and Ser261 in PCNA-3 in the cracked-PCNA intermediate 2 (Fig. 6f). Owing to the numerous RFC5–PCNA interactions, we suggest that movements of the RFC5 AAA+ module control PCNA, converting it between a cracked spiral and a planar ring.

### ATAD5-RFC opens a different gap in PCNA than RFC

**Fig. 7:**
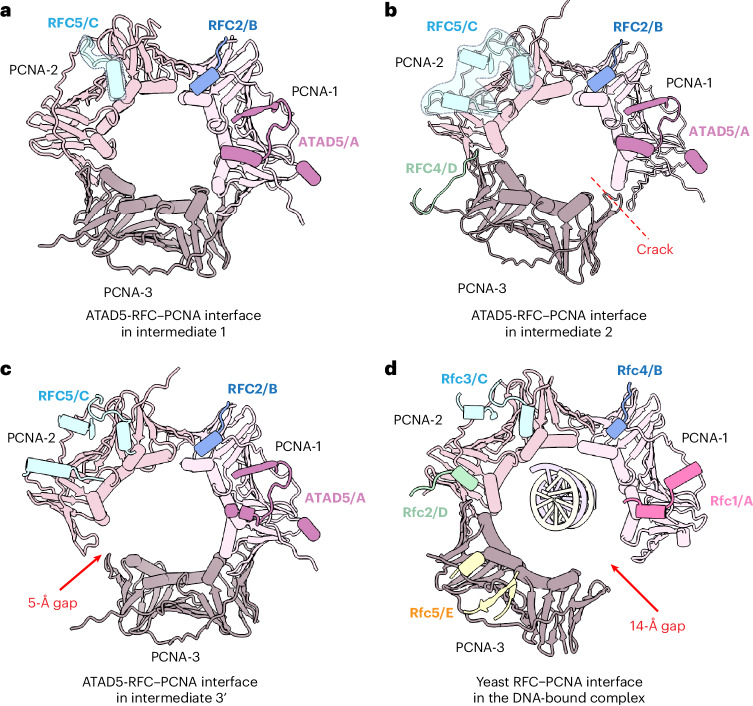
The PCNA ring is opened by ATAD5-RFC and by RFC at different locations. **a**-**c**, Top views of the interfaces between ATAD5-RFC and the PCNA ring in intermediate states 1 (**a**), 2 (**b**) and 3′ (**c**). Structures are shown in cartoons and colored by individual subunits. The structural elements of the ATD5–RFC subunits contacting the PCNA are labeled. The red arrow in **c** points to the 5-Å gap between PCNA-2 and PCNA-3. **d**, Top view of the interface between yeast RFC and PCNA (PDB: 7TFI). All RFC subunits contact the PCNA ring. The red arrow points to the 14-Å gap between PCNA-3 and PCNA-1. PCNA-3 is in the lowest position in ATAD5-RFC–PCNA (**c**), but is in the highest position in RFC–PCNA (**d**).

In the planar-PCNA intermediate state 1, the interactions involving PCNA-1 remain the same, but the N terminus of RFC5 and the shorter α-helix lose contact with PCNA-2, and no subunit is in contact with PCNA-3 (Fig. 7a). In the cracked-PCNA intermediate state 2, ATAD5 contacts the middle of PCNA-1, RFC2 (subunit B) contacts the interface between PCNA-1 and PCNA-2, RFC5 (subunit C) contacts PCNA-2, and an RFC4 (subunit D) loop weakly binds PCNA-3 (Fig. 7b). In the open-PCNA intermediate state 3′, there is a 5-Å gap between PCNA-2 and PCNA-3 (the PCNA-3/2 gap). PCNA homotrimer exhibits a spiral shape in both the cracked-PCNA and open-PCNA states 2 and 3′, but only in the open-PCNA intermediate 3′ is PCNA-3 free to move further down to enlarge the gap (Fig. 7c). There is no PCNA-3/1 gap in all three observed ATAD5-RFC intermediate states.

It is unclear whether the observed PCNA-3/2 gap is used by ATAD5-RFC to unload PCNA from DNA. If the PCNA-3/2 gap is used for PCNA unloading, it would be different from PCNA-3/1 gap that is used for PCNA loading^17,18^ (Fig. 7d). This scenario is plausible because there is no need for ATAD5-RFC to open one particular gap in PCNA. This is unlike RFC, which must open the PCNA-3/1 gap to vertically align the PCNA gap with the A-gate in the loader for lateral DNA entry into both PCNA and RFC at the same time. By contrast, the ATAD5-RFC A-gate is locked shut, and DNA does not enter the central chamber, so there is no need for ATAD5-RFC to open the PCNA-3/1 gap, which is situated below the A-gate. Therefore, the PCNA-2/3 gap could possibly be used for PCNA unloading.

We propose that all the structures observed here are post-unloading states. As the most prevalent conformer, especially after a 20-min reaction, the planar-PCNA intermediate state 1 might be the most stable of the intermediate states. It has been documented that clamp loaders expend binding energy to open a clamp, and therefore the clamp loader binds tighter to a clamp with a mutant interface, implying that the affinity of the clamp loader is lower for an opened WT clamp^39,41^. This seems to also hold true for the interaction between ATAD5-RFC and PCNA^31^. Although we cannot be certain of the order of these intermediate steps, we suggest two possible scenarios. In one scenario, the cracked-PCNA intermediate 2 could occur just after the release of PCNA from DNA, followed by ATP hydrolysis to yield the disordered and open intermediates 3 and 3′, which should have lower affinity to, and most easily disengage from, PCNA owing to expenditure of binding energy (between ATAD5-RFC and PCNA) used to open PCNA. Then, ATAD5-RFC may exchange ADP for ATP in RFC4 (subunit D) and preferentially bind closed PCNA to form the complex with the lowest free energy, planar-PCNA intermediate 1. Alternatively, after PCNA unloading (driven by ATP binding), ATP is hydrolyzed in RFC4 to form the disordered and open-PCNA intermediates 3 and 3′, followed by ADP exchange for ATP to yield the cracked-PCNA intermediate 2 and then the planar-PCNA intermediate 1.

## Discussion

After Okazaki fragment synthesis and nick ligation, PCNA needs to be unloaded^42^. The PCNA loaders have been shown to also possess PCNA-unloading activity, and we show here that ATAD5 has evolved unique features that explain why it is a dedicated unloader. Key features include the long LL1 of ATAD5 and the chamber plug, both of which appear to rigidify the ATAD5-RFC complex and lock the A-gate shut. Therefore, instead of admitting DNA into its chamber, ATAD5-RFC can only push DNA out of the PCNA ring, thereby unloading PCNA. These key structural features are also present in the recently reported structure of the yeast PCNA unloader Elg1-RFC^43^. Therefore, the overall strategy of converting a clamp loader to a clamp unloader is similar between yeast and humans. Furthermore, both Elg1-RFC and ATAD5-RFC were observed binding to a planar PCNA as well as a cracked PCNA, suggesting that the PCNA-unloading mechanism is conserved in eukaryotes.

Notably, we have captured an unprecedented intermediate of ATAD5-RFC in which both RFC4 (subunit D) and RFC3 (subunit E) are bound to ADP, in addition to the expected intermediates shared among all clamp loaders and unloaders in which only RFC3 (subunit E) is bound to ADP and all other subunits are bound to ATPγS. PCNA-3 is made highly mobile by ATAD5-RFC in the intermediate with two ADP molecules, and a PCNA-3/2 gap can form. The location of this gap is different from the PCNA gap opened by all structurally characterized clamp loaders that invariably open PCNA between the PCNA-3 and PCNA-1 subunits. What’s more, the A-gate opening in the clamp loaders corresponds to the PCNA gap opening between PCNA-3 and PCNA-1. However, structural comparisons between the ATAD5-RFC–PCNA complex and the RFC–PCNA complex show that ATAD5-RFC cannot open the A-gate (Extended Data Fig. 5c–e), suggesting that ATAD5-RFC is unable to open the PCNA-3/1 gap like RFC clamp loaders can. Thus, it is possible that the clamp-unloading mechanism is fundamentally distinct from the clamp-loading mechanism. Although we included a DNA substrate in the in vitro assembly and used the well-established procedure that allows DNA to thread into PCNA in vitro^38^, no DNA-bound unloading intermediates were captured in this study. Thus, the detailed unloading mechanism remains unknown and requires further investigation.

## Methods

### Generation of DNA constructs

Human ATAD5 complementary DNA was purchased from DNASU Plasmid Repository, and the cDNAs encoding all other components of the human ATAD5-RFC complex (RFC2–RFC5) were purchased from Addgene. The N-terminal truncated human ATAD5 (812–end) cDNA was tagged with an N-terminal 3×FLAG and a C-terminal 6×His tag and cloned into the pFast-Bac Dual Expression vector (Thermo Fisher Scientific). The cDNA of RFC2, RFC3, RFC4 and RFC5 was cloned into the pFL multi-gene expression vector pLIB_RFC2-5, following the protocol for biGBac, with minor modifications^45^. To produce the human RFC and the PCNA complexes, the cDNAs of the N-terminal truncated human RFC1 (∆N555) and PCNA were cloned into the pET28a vector with an N-terminal 6×His tag and a thrombin cleavage site, respectively. All constructs were sequenced to ensure that no mutations were introduced during PCR and cloning.

### Protein expression and purification

To express ATAD5-RFC, the Bac-to-Bac Baculovirus expression system (Thermo Fisher Scientific) was used. Sf9 or Hi5 cells (5 × 10^5^ cells ml^–1^) were coinfected with two baculoviruses; one baculovirus encoded ATAD5 with an N-terminal 3× FLAG tag and thrombin cleavage site (as described above), and the other encoded RFC2–RFC5. Coinfected cells were incubated at 27 °C for 72 h with gentle shaking (115 r.p.m.). ATAD5-RFC was purified from infected insect cells upon lysis by sonication in lysis buffer (25 mM HEPES, pH 7.5, 250 mM NaCl, 1 mM magnesium acetate and 1 EDTA-free protease inhibitors cocktail tablet). The lysate was clarified by centrifugation at 125,440*g* for 1 h at 4 °C using a Ti-45 rotor. The clarified supernatant was then incubated with 0.8 ml FLAG-antibody-coated beads at 4 °C for 2–3 h. Beads were washed with 50 ml lysis buffer, and proteins were eluted with 8 ml lysis buffer containing 0.2 mg ml^–1^ 3× FLAG peptide. Proteins were concentrated using centrifugal concentrators (Amicon, 100 kDa) and further purified by size-exclusion chromatography (SEC, Superose 6 Increase, GE Healthcare) in 25 mM HEPES, pH 7.5, 200 mM NaCl, 1 mM magnesium acetate and 1 mM DTT. Purified protein was concentrated to 3.2 mg ml^–1^ and stored at –80 °C.

Human PCNA (hPCNA) used for cryo-EM was expressed in and purified from *E. coil* BL21. To purify hPCNA from *E. coil* cells, transformants were grown at 37 °C until the cell density reached an optical density at 600 nm of 0.8, then 0.2 mM isopropyl-β-d-thiogalactopyranoside (IPTG) was added to induce protein expression. The culture was induced for 12 h at a reduced temperature of 16 °C. Cells were collected by centrifugation at 4 °C, resuspended in buffer A (25 mM HEPES, pH 7.5, 200 mM NaCl, 1 mM magnesium acetate) and lysed using a homogenizer (SPX Corporation). The lysate was clarified by centrifugation at 34,572*g* for 1 h at 4 °C, and the supernatant was applied to a 5-ml Ni-NTA affinity column (Cytiva). Protein was eluted using buffer A plus 300 mM imidazole. The N-terminal 6×His-tag was removed using thrombin at 4 °C overnight, and was then subjected to size-exclusion chromatography using a Superdex 200 column (GE Healthcare) in 20 mM HEPES, pH 7.5, 200 mM NaCl, 1 mM magnesium acetate and 1 mM DTT. Human RFC was overexpressed and purified in *E. coli*, as previously described^15^. The pET28-hRFC1∆N555 and pET-Duet-p36-p37-p38-p40 plasmids were cotransformed into BL21(DE3) for expression and purification of the RFC complex.

### PCNA-unloading reactions

Clamp-unloading reactions were performed in two steps. First ^32^P-labaled PCNA was loaded onto a nicked circular plasmid by RFC and then gel filtered to remove the RFC and free ^32^P-labeled PCNA. The purified ^32^P-PCNA–DNA was then incubated with ATAD5-RFC (or RFC) and gel filtered a second time to separate ^32^P-labeled PCNA–DNA complex from ^32^P-labeled PCNA that had been unloaded from the DNA. The proteins were purified as follows: hPCNA was tagged with both a hexahistidine and six-residue kinase site, as previously described^46^, and then purified from *E. coli*, as previously described^14^. The PCNA was radiolabeled with ^32^P, as previously described^46^, and the molar specific activity of the ^32^P-labeled PCNA was determined through scintillation counting and Bradford protein stain (Bio-Rad). Human RFC, lacking the 555 N-terminal residues of Rfc1, was expressed in *E. coli* and purified as previously described^47^. The ATAD5-RFC (WT and five mutants) were purified from SF9 cells, as described in this report. To test ATAD5-RFC for nuclease activity, 500 ng DNA and 22 nM ATAD5-RFC were incubated at 37 °C in 20 μl of 20 mM Tris-Cl, pH 7.5, 0.5 mM EDTA, 4% glycerol, 0.1 mg ml^–1^ BSA, 8 mM MgCl_2_, 2 mM DTT, 2 mM ATP and 100 mM NaCl for 5 or 20 min, then quenched with SDS-loading dye. Reactions were analyzed on a 0.8% native agarose gel in TBE buffer (89 mM tris-borate, pH 8.3, 2 mM EDTA) containing ethidium bromide.

The initial clamp-loading reaction contained 3 pmol Nt-BspQI-nicked pUC19, 6 pmol ^32^P-labeled PCNA and 2.4 pmol RFC in 200 μl buffer A containing 2 mM ATP. Reactions were incubated at 37 °C for 15 min, then applied to a 5 ml BioGel A15m column equilibrated in buffer A containing 100 mM NaCl. Seven drop fractions (approximately 200 μl each) were collected. DNA-bound ^32^P-labeled PCNA eluted in fractions 11–16, whereas the unbound free ^32^P-labeled PCNA eluted later (mainly fractions 20–30). Fractions containing ^32^P-labeled PCNA–DNA were combined for use in unloading assays.

The PCNA-unloading assay contained 420 fmol ^32^P-labeled PCNA–nicked DNA complex and 2 pmol ATAD5-RFC or RFC in 200 μl buffer A with 2 mM ATP and 100 mM NaCl. Reactions were incubated at 37 °C for 5 min before separation on a 5-ml BioGel A15m column using buffer A + 100 mM NaCl. Seven drop fractions were collected, and radioactivity was measured in 180-μl aliquots using liquid scintillation counting. The specific radioactivity of ^32^P-labeled was used to convert counts per minute into femtomoles ^32^P-labeled PCNA.

### Preparation of ATAD5-RFC–PCNA(–DNA) complexes for cryo-EM

The dsDNA substrates were chemically synthesized by Eurofins Genomics and included a 38-nt Watson strand (5′-TCTTCTTTCACTGCCCTTTATTTATAAGACTCATGTCC-3′) and a 38-nt Crick strand (5′-GGACATGAGTCTTATAAATAAAGGGCAGTGAAAGAAGA-3′). The DNA oligonucleotides were annealed by mixing at a final concentration of 100 μM in annealing buffer (20 mM HEPES, pH 7.5, 50 mM NaCl and 0.5 mM EDTA) and were subjected to heat denaturation at 95 °C for 10 min, and the temperature was then gradually decreased to room temperature.

To reconstitute the PCNA-unloading intermediates by the ATAD5-RFC in vitro, 1.5 μl purified hPCNA (90 μM) and 4.5 μl annealed DNA substrate (100 μM) were mixed and incubated at 30 °C for 10 min. This was followed by the addition of 7.25 μl ATAD5-RFC (6 μM) with 0.75 μl ATPγS (10 mM) and 1 μl magnesium acetate (100 mM). The final concentrations of these components in the 15-μl reaction volume were: 2.9 μM ATAD5-RFC, 3 μM PCNA (as trimer), 30 μM annealed DNA substrate, 0.5 mM ATPγS and 6.7 mM magnesium acetate. This was equivalent to an approximate molar ratio of 1 (ATAD5-RFC):1 (PCNA clamp):10 (DNA). The mixture was then incubated in an ice-water bath for 20 min before cryo-EM grids were prepared. In such a reaction mixture, only the ATAD5-RFC bound to a closed PCNA was observed in EM images. In attempts to capture ATAD5-RFC in the process of unloading DNA-bound PCNA, the reaction mixture was incubated for shorter periods (10 min, 6 min or 3 min). However, no DNA-bound complex was observed. This observation suggests that DNA is pushed out of the PCNA ring quickly, probably as soon as ATAD5-RFC engages the clamp. Finally, in the mixture that was incubated for 3 min, we determined three ATAD5-RFC–PCNA structures in which the PCNA ring was differently cracked.

To verify the prepared PCNA–DNA complex, the diluted PCNA–DNA mixture was examined in cryo-EM. hPCNA alone was also incubated with ATAD5-RFC for 10 min in the same buffer and at the same concentration as that used for the PCNA-unloading intermediates by ATAD5-RFC.

### ATPase assays

The ATP hydrolysis rate of ATAD5-RFC incubated with PCNA, DNA or a PCNA–DNA mixture was measured using the Malachite Green Phosphate Assay Kit (Sigma-Aldrich). ATPase reactions were conducted at room temperature in a reaction buffer containing 25 mM HEPES pH 7.6, 2 mM MgCl_2_, 100 mM NaCl and 1 mM DTT; 0.25 μM ATAD5-RFC was incubated for 3 min with 0.75 μM PCNA, 0.75 μM DNA or 0.75 μM PCNA and 0.75 μM DNA mixture in the presence of 1 mM ATP. The reactions were stopped by the addition of 1% SDS and 50 mM EDTA. Absorbance at 620 nm was measured in a SpectraMax M2e microplate reader (Molecular Devices). The ATPase activity was calculated using Microsoft Excel.

### Preparation of cryo-EM grids and data collection

Holey carbon grids (Quantifoil Au R2/1, 400 gold mesh) were used. Before samples were cryogenically frozen on grids, we freshly glow-discharged the EM grids in ArO_2_ for 30 s using a Gatan 950 Solarus plasma-cleaning system, with the power source set to 15 W. We first pipetted 3 μl of the PCNA–DNA mixture on EM grids, then pipetted another 3-μl droplet of the prepared ATAD5-RFC and PCNA–DNA mixture (or PCNA alone). Grids were blotted for 3 s after each sample application with a blotting force set to 3, then flash-frozen in liquid ethane using an FEI Vitrobot Mark IV. Relative humidity and temperature inside the blotting chamber were set to 100% and 6 °C, respectively. Grids were initially checked on a 200 kV Talos Arctica electron microscope, and high-quality datasets were collected automatically in the multi-hole mode on a 300 kV Titian Krios electron microscope controlled by SerialEM^48^. Images were collected at ×105,000 using an objective lens defocus range of –1.2 to –1.8 µm. Micrograph images were recorded using a K3 direct electron detector (Gatan) operated in super-resolution video mode, corresponding to a pixel size of 0.414 Å at specimen level. A total of 50 frames for each micrograph were recorded with a total exposure time of 1.0 s and total dose of 60 e^–^/Å^2^.

### Image processing and 3D reconstruction

For 20-min unloading reactions, we collected 18,828 raw video micrographs using the Titan Krios EM and performed motion-correction on each micrograph using the program MotionCorr-2.0 (ref. ^49^) with 2× binning, resulting in a pixel size of 0.828 Å (Extended Data Fig. 3). We then imported the micrographs into cryoSPARC^50^ (version 4.2.3) for patch-based contrast transfer function (CTF) estimation and correction. A total of 17,862 micrographs with CTF signals extending to 4.0 Å were retained for further processing. We first used blob-based auto-picking (90–150 Å diameter) in cryoSPARC to select initial particle images and generate 2D templates, and then used the templates for another round of particle picking. In total, 2,157,746 particles were extracted. We then performed three rounds of 2D classifications on 4× binned particle images to obtain ‘particle classes’ with shared features, and retained particles in classes with clear structural features. We next used 1,000,114 particles that were extracted using a box size of 320 pixels to calculate five starting 3D models. Four 3D reconstructions were discarded because they lacked structural details (for example, partial complexes or junk particles). The major class of particles (48%) in the 3D reconstruction was chosen for heterogeneous refinement, leading to three 3D classes. The 3D class containing the most particles (>60% of the dataset) had the best structural details. Particles in this 3D class were re-extracted from micrographs of the original pixel size and subjected to homogeneous and non-uniform refinements, resulting in a 3D map at an overall resolution of 2.97 Å. Because the PCNA density was weak in the EM map, we performed further heterogeneous refinement, leading to three 3D classes, of which two 3D classes with the most particles, and at higher resolution, were selected: particles in these two 3D classes were subjected to another round of homogeneous and non-uniform refinements, leading to the final 3D map with improved PCNA density at of 3.04-Å overall resolution.

For the 3-min unloading reaction, we collected 20,810 micrographs using the Titan Krios. The initial MotionCorr-2 and CTF refinement were performed similarly as described for the 20-min dataset, using selected 2D classes from the 20-min dataset for template picking (Extended Data Fig. 8). A total of 20,810 micrographs with CTF signals extending to 4.0 Å were retained, and 2,086,937 particles were extracted. We then performed two rounds of 2D classifications on 4× binned particle images, and selected particles belonging to 2D classes with clear structural features. We extracted 1,027,202 particles with a box size of 320 pixels and used these particles to calculate six initial 3D models. Among the six maps, four 3D reconstructions were discarded because they contained too few particles or had partial (broken) complexes; a 3D reconstruction (31.9% of particles) with a gapped PCNA ring was chosen for further homogeneous and non-uniform refinements, leading to a 3D map with an overall resolution of 3.1 Å. Another 3D reconstruction containing 24.8% of particles (that is, the cracked PCNA ring) was subjected to further heterogeneous refinement, resulting in three 3D classes. The major 3D class had the best resolution and was selected for another round of homogeneous and non-uniform refinements, leading to the final 3D map, which had an overall resolution of 3.48 Å.

One PCNA subunit was invisible in the 3.1-Å EM map with a gapped PCNA ring, indicating that this PCNA region is very flexible. Therefore, we performed 3DVA^51^ to investigate the conformational variation of the PCNA ring. The final dataset with 327,296 particle images was 2× binned and subjected to non-uniform refinement in *C*_1_ symmetry to generate an appropriate mask. 3DVA was performed to obtain three subclasses with the filter resolution set to 8 Å; all other parameters were set to default. The distribution of reaction coordinates was smooth across particles in the principal components, confirming the compositional homogeneity of the selected dataset and the conformational flexibility of the PCNA ring. One 3DVA subclass with a uniquely cracked PCNA ring that was not observed in other EM maps was selected for further processing. This subclass contained 55,855 particles, and homogeneous and non-uniform refinements with the subclass dataset led to a 3D map at an overall resolution of 4.30 Å.

A similar workflow was used to obtain clear 2D class average images of the Arctica datasets. In the EM dataset of the mixture of ATAD5-RFC and PCNA (without DNA substrate), there were sufficient particles for 3D classification. A good 3D class was selected for homogeneous and non-uniform refinement, leading to a 3D map at an overall resolution of 4.20 Å (Extended Data Fig. 8). This map of ATAD5-RFC–PCNA is very similar to the 3D map obtained from the 20-min incubated unloading mixture sample that contained the DNA substrate.

### Model building, refinement and validation

The structures of human ATAD5, RFC2–RFC5 and human PCNA predicted by AlphaFold2 (ref. ^52^), combined with the previously reported structure of the human RFC–PCNA complex (PDB: 6VVO), were used for atomic model building in the four EM maps of ATAD5-RFC and PCNA. The human RFC–PCNA model was fitted into the EM map of the ATAD5-RFC–closed PCNA; in the AlphaFold model of ATAD5, the flexible loop was removed and replaced with RFC1 to generate the starting model in UCSF ChimeraX^53^. This model was manually rebuilt in Coot to include the missed residues in the plug region of ATAD5 and the terminal residues in RFC2–RFC5 and PCNA. The models were real-space refined in PHENIX^54^. For the LL1 with weaker EM densities, the AlphaFold-Multimer server^55^ was used to predict the complex structure of LL1 with RFC2 and RFC5. All predicted complex structures had a high confidence value with a consistent interface between LL1 and RFC2–RFC5. And the predicted complex structure fitted well in the EM densities in the LL1, RFC2 and RFC5 regions in the ATAD5-RFC–closed PCNA map. Through several rounds of real-space refinement in PHENIX and manual building in coot, the atomic model of the ATAD5-RFC–closed PCNA complex was refined to 3.2 Å. The refined model was validated by the MolProbity program embedded in PHENIX^56,57^. Then, the ATAD5-RFC–closed PCNA model was used as a starting model for modeling building into the three EM maps of the ATAD5-RFC–cracked PCNA (3.48 Å) and ATAD5-RFC with gapped PCNA ring (3.10 Å and 4.20 Å). These models were refined in PHENIX and manually adjusted and rebuilt to fit the EM densities in Coot, following a similar process to the one described above. The final atomic models were validated using MolProbity^56^. Model-refinement statistics are shown in Table 1. The original EM maps were sharpened using the deepEMhancer^44^ to prepare figures. All structural figures were prepared in the UCSF ChimeraX^53^.

### Reporting summary

Further information on research design is available in the Nature Portfolio Reporting Summary linked to this article.

## Online content

Any methods, additional references, Nature Portfolio reporting summaries, source data, extended data, supplementary information, acknowledgements, peer review information; details of author contributions and competing interests; and statements of data and code availability are available at 10.1038/s41594-024-01332-4.

## Supplementary information

Reporting Summary

Supplementary Video 1 Three-dimensional variability analysis of intermediate state 3 of the human ATAD5-RFC–gapped PCNA complex reveals the dramatic up-and-down movement of the PCNA-3 subunit.

## Source data

Source Data Fig. 3 and Extended Data Figs. 1 and 2 Unmodified gels.

Source Data Fig. 3 PCNA-unloading assay data points (3g).

Source Data Extended Data Fig. 2 Source data of ATPase assay (2b) and PCNA-unloading assay (2d, 2e, 2f).

## Data Availability

The 3D cryo-EM maps of the human ATAD5 bound to the PCNA ring have been deposited in the EMDB under accession codes EMD-42295 (ATAD5-RFC–closed PCNA, 3.04 Å), EMD-42289 (ATAD5-RFC–cracked PCNA, 3.48 Å), EMD-42288 (ATAD5-RFC–gapped PCNA, 3.10 Å) and EMD-42287 (ATAD5-RFC–gapped PCNA, 4.20 Å). Their corresponding atomic models have been deposited in the Protein Data Bank under accession codes 8UII, 8UI9, 8UI8 and 8UI7, respectively. All experimental data are available upon reasonable request. Source data are provided with this paper.
