# Supplementary material for: The human ATAD5 has evolved unique structural elements to function exclusively as a PCNA unloader
Source: Nat Struct Mol Biol. 2024 Jun 13;31(11):1680–91. doi: 10.1038/s41594-024-01332-4 (PMC11563871; doi:10.1038/s41594-024-01332-4)
Supplement: Supplementary file 1 — Reporting Summary [file 41594_2024_1332_MOESM1_ESM.pdf]

nature portfolio

Corresponding author(s): Huilin Li

Last updated by author(s): Apr 17, 2024

Reporting Summary

Nature Portfolio wishes to improve the reproducibility of the work that we publish. This form provides structure for consistency and transparency in reporting. For further information on Nature Portfolio policies, see our Editorial Policies and the Editorial Policy Checklist.

Statistics

For all statistical analyses, confirm that the following items are present in the figure legend, table legend, main text, or Methods section.

n/a	Confirmed	
☐	☒	The exact sample size (*n*) for each experimental group/condition, given as a discrete number and unit of measurement
☐	☒	A statement on whether measurements were taken from distinct samples or whether the same sample was measured repeatedly
☒	☐	The statistical test(s) used AND whether they are one- or two-sided<br>*Only common tests should be described solely by name; describe more complex techniques in the Methods section.*
☐	☒	A description of all covariates tested
☐	☒	A description of any assumptions or corrections, such as tests of normality and adjustment for multiple comparisons
☐	☒	A full description of the statistical parameters including central tendency (e.g. means) or other basic estimates (e.g. regression coefficient) AND variation (e.g. standard deviation) or associated estimates of uncertainty (e.g. confidence intervals)
☒	☐	For null hypothesis testing, the test statistic (e.g. *F*, *t*, *r*) with confidence intervals, effect sizes, degrees of freedom and *P* value noted<br>*Give P values as exact values whenever suitable.*
☒	☐	For Bayesian analysis, information on the choice of priors and Markov chain Monte Carlo settings
☒	☐	For hierarchical and complex designs, identification of the appropriate level for tests and full reporting of outcomes
☒	☐	Estimates of effect sizes (e.g. Cohen's *d*, Pearson's *r*), indicating how they were calculated

Our web collection on statistics for biologists contains articles on many of the points above.

Software and code

Policy information about availability of computer code

Data collection	SeriEM (https://bio3d.colorado.edu/SerialEM/) was used to collect Cryo-EM micrographs;<br>SpectraMax M2e microplate reader was used to test the ATPase activity;<br>Liquid scintillation counting is used to perform the in vitro PCNA unloading assay.
Data analysis	UCSF ChimeraX (version 1.6) were used to prepare figures of cryo-EM maps and atomic models.<br>Relion4 (https://relion.readthedocs.io/en/release-4.0/) and Cryosparc (https://cryosparc.com, version 4.2) were used for cryo-EM image processing. AlphaFold-Multimer server was used to build the initial atomic model.<br>Phenix (version 1.21-5207) and Coot (version 0.9.8.7) were used for model building and refining.<br>MolProbity (version 4.5.2)for model validation, deepEMhancer for map sharpening.<br>Microsoft Excel for Microsoft 365 was used for the ATPase activity analysis.

For manuscripts utilizing custom algorithms or software that are central to the research but not yet described in published literature, software must be made available to editors and reviewers. We strongly encourage code deposition in a community repository (e.g. GitHub). See the Nature Portfolio guidelines for submitting code & software for further information.

Data

Policy information about availability of data

All manuscripts must include a data availability statement. This statement should provide the following information, where applicable:

- Accession codes, unique identifiers, or web links for publicly available datasets
- A description of any restrictions on data availability
- For clinical datasets or third party data, please ensure that the statement adheres to our policy

The 3D cryo-EM maps of the human ATAD5 bound the PCNA ring have been deposited in Electron Microscopy Data Bank (EMDB) under accession codes EMD-42295 (ATAD5-RFC–closed PCNA, 3.04 Å), EMD-42289 (ATAD5-RFC–cracked PCNA, 3.48 Å), EMD-42288 (ATAD5-RFC–gapped PCNA, 3.10 Å), EMD-42287 (ATAD5-RFC–gapped PCNA, 4.3 Å), respectively. Their corresponding atomic models have been deposited in Protein Data Bank under accession codes 8UII, 8UI9, 8UI8, 8UI7, respectively.
The raw data for PCNA unloading assay and the ATPase assay of ATAD5-RFC and mutants were provided as the source data.

Research involving human participants, their data, or biological material

Policy information about studies with human participants or human data. See also policy information about sex, gender (identity/presentation), and sexual orientation and race, ethnicity and racism.

Reporting on sex and gender	n/a
Reporting on race, ethnicity, or other socially relevant groupings	n/a
Population characteristics	n/a
Recruitment	n/a
Ethics oversight	n/a

Note that full information on the approval of the study protocol must also be provided in the manuscript.

Field-specific reporting

Please select the one below that is the best fit for your research. If you are not sure, read the appropriate sections before making your selection.

☒ Life sciences ☐ Behavioural & social sciences ☐ Ecological, evolutionary & environmental sciences

For a reference copy of the document with all sections, see nature.com/documents/nr-reporting-summary-flat.pdf

Life sciences study design

All studies must disclose on these points even when the disclosure is negative.

Sample size	No astatistical analyses were used to determine the appropriate sample size. For Cryo-EM analysis, over 10,000 micrographs were collected in each dataset, and millions of particles were picked to preform a homogenous 3D reconstruction.
Data exclusions	Low quality particle images were excluded based on CTF signal and 2D and 3D classifications. These are standard practice in cryo-EM to use only the very best data for final 3D reconstruction and refinement.
Replication	Reproducibility lies in the large number of particle images used in 3D reonstruction, as well as in the validation report provided by the PDB data bank. All ATPase activity assaies were performed independently at least three times. The PCNA unloading assay were performed twice though gel filtration. All biochemical assay data were replicated successfully.
Randomization	Data (2D images or 3D EM maps) were visible to the researchers and were not randomized. Randomization is not needed in this kind of cryo-EM research. Randomization is less relevant to other in vitro biochemical analysis because the biochemical assay was designed by the precise condition and mutants.
Blinding	Blinding is not required for this type of research. All the in vitro biochemical experiments have standardized protocols which are designed to minimize variability and ensure consistency of experimental conditions.

Reporting for specific materials, systems and methods

We require information from authors about some types of materials, experimental systems and methods used in many studies. Here, indicate whether each material, system or method listed is relevant to your study. If you are not sure if a list item applies to your research, read the appropriate section before selecting a response.

Materials & experimental systems

n/a	Involved in the study
☒	☐ Antibodies
☐	☒ Eukaryotic cell lines
☒	☐ Palaeontology and archaeology
☒	☐ Animals and other organisms
☒	☐ Clinical data
☒	☐ Dual use research of concern
☒	☐ Plants

Methods

n/a	Involved in the study
☒	☐ ChIP-seq
☒	☐ Flow cytometry
☒	☐ MRI-based neuroimaging

Eukaryotic cell lines

Policy information about cell lines and Sex and Gender in Research

Cell line source(s)	Spodoptera frugiperda Sf9 RV free cells were purchased from Expression Systems.
Authentication	The commercial cell line from ThermoFisher Scientific (https://www.thermofisher.com/us/en/home/references/gibco-cell-culture-basics/cell-morphology/morphology-of-sf9-cells.html)
Mycoplasma contamination	Negative
Commonly misidentified lines (See ICLAC register)	No commonly misidentified cell lines were used in this study.

Plants

Seed stocks	n/a
Novel plant genotypes	n/a
Authentication	n/a
