# Supplementary material for: The human ATAD5 has evolved unique structural elements to function exclusively as a PCNA unloader
Source: Nat Struct Mol Biol. 2024 Jun 13;31(11):1680–91. doi: 10.1038/s41594-024-01332-4 (PMC11563871; doi:10.1038/s41594-024-01332-4)

Entire SDS-PAGE for Figure 3f

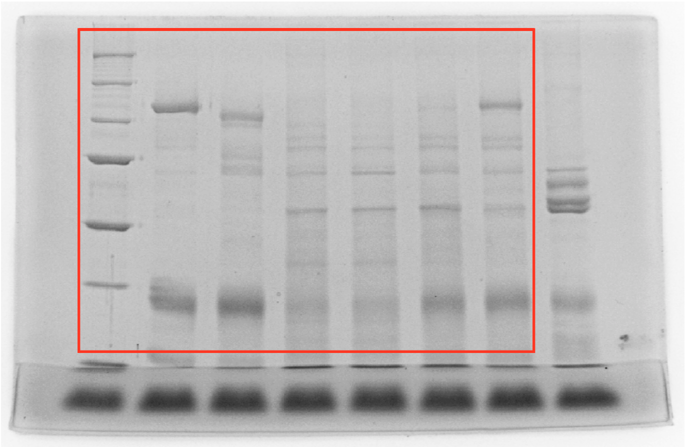

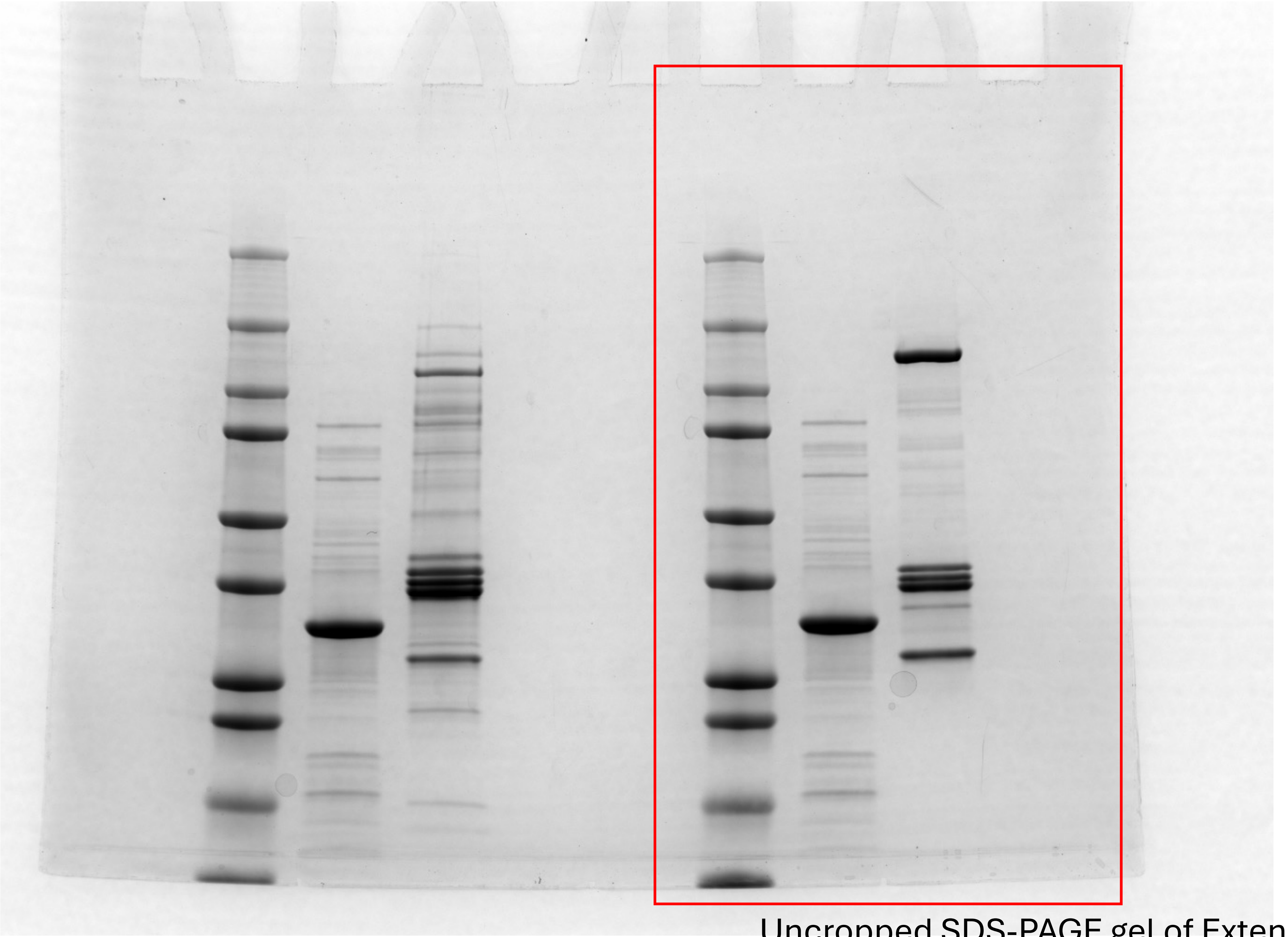

Uncropped SDS-PAGE gel of Extend Data Fig. 1a

Entire SDS-polyacrylamide gel for Supp. Figure 2a

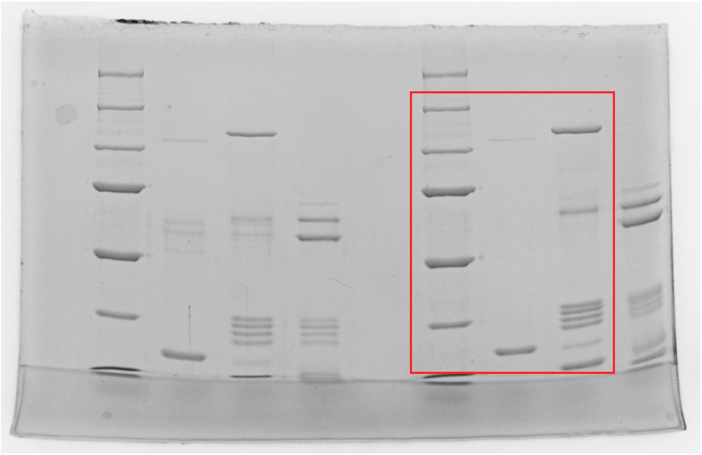

Entire agarose gel for Supp. Figure 2c

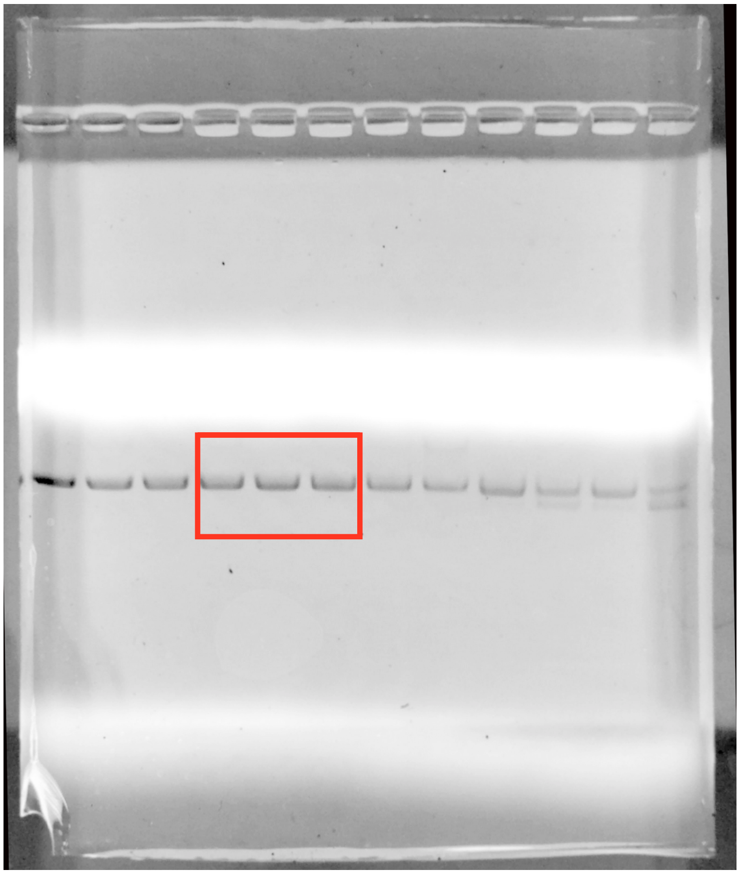

Supplement: Supplementary file 3 — Unmodified gels. [file 41594_2024_1332_MOESM3_ESM.pdf]
